# Pt(II)-PLGA Hybrid in a pH-Responsive Nanoparticle System Targeting Ovarian Cancer

**DOI:** 10.3390/pharmaceutics15020607

**Published:** 2023-02-10

**Authors:** Marek T. Wlodarczyk, Sylwia A. Dragulska, Ying Chen, Mina Poursharifi, Maxier Acosta Santiago, John A. Martignetti, Aneta J. Mieszawska

**Affiliations:** 1 Department of Chemistry, Brooklyn College, The City University of New York, 2900 Bedford Avenue, Brooklyn, NY 11210, USA; 2 Ph.D. Program in Chemistry, The Graduate Center of the City University of New York, New York, NY 10016, USA; 3 Department of Genetics and Genomic Sciences, Icahn School of Medicine at Mount Sinai, 1425 Madison Avenue, New York, NY 10029, USA; 4 Ph.D. Program in Biochemistry, The Graduate Center of the City University of New York, New York, NY 10016, USA; 5 Women’s Health Research Institute, Icahn School of Medicine at Mount Sinai, 1 Gustave L. Levy Pl, New York, NY 10029, USA; 6 Rudy Ruggles Research Institute, Western Connecticut Health Network, 131 West St., Danbury, CT 06810, USA

**Keywords:** nanoparticles, pH-sensitive, ovarian cancer, platinum therapy, in vivo imaging

## Abstract

Platinum-based agents are the main treatment option in ovarian cancer (OC). Herein, we report a poly(lactic-co-glycolic acid) (PLGA) nanoparticle (NP) encapsulating platinum (II), which is targeted to a cell-spanning protein overexpressed in above 90% of late-stage OC, mucin 1 (MUC1). The NP is coated with phospholipid-DNA aptamers against MUC1 and a pH-sensitive PEG derivative containing an acid-labile hydrazone linkage. The pH-sensitive PEG serves as an off–on switch that provides shielding effects at the physiological pH and is shed at lower pH, thus exposing the MUC1 ligands. The pH-MUC1-Pt NPs are stable in the serum and display pH-dependent PEG cleavage and drug release. Moreover, the NPs effectively internalize in OC cells with higher accumulation at lower pH. The Pt (II) loading into the NP was accomplished via PLGA-Pt (II) coordination chemistry and was found to be 1.62 wt.%. In vitro screening using a panel of OC cell lines revealed that pH-MUC1-Pt NP has a greater effect in reducing cellular viability than carboplatin, a clinically relevant drug analogue. Biodistribution studies have demonstrated NP accumulation at tumor sites with effective Pt (II) delivery. Together, these results demonstrate a potential for pH-MUC1-Pt NP for the enhanced Pt (II) therapy of OC and other solid tumors currently treated with platinum agents.

## 1. Introduction

Platinum (II) therapy, e.g., cisplatin, carboplatin, and oxaliplatin, is broadly used to treat many malignancies, including testicular, bladder, and ovarian cancer [1,2]. Ovarian cancer (OC) is the leading cause of gynecologic cancer death and is usually diagnosed as a late-stage disease [3,4,5]. The gold standard treatment consists of debulking surgery combined with platinum/taxane combination therapy. Although initial clinical response rates are ~70–80%, recurrence rates resulting from platinum resistance are high, leading to overall reduced survival rates [3,6]. Pt (II) complexes bind to nuclear DNA, interfering with DNA duplication and transcription, which eventually induces cancer cell apoptosis [1,7]. Although Pt (II) complexes are among the most potent anti-cancer agents used in the clinic, severe side effects compromise the benefit of platinum therapy [8]. Therefore, low clinical dosages of Pt (II) are used, often leading to subtherapeutic intracellular concentrations of the drug [9]. The insufficient DNA damage and activation of DNA repair mechanisms, such as the nucleotide excision repair responsible for platinum-DNA adduct removal, are considered the primary mediators of platinum resistance [10,11].

Nanoparticles (NPs) offer unique shielding effects and sustained release that can potentially diminish the severe side effects of highly toxic agents [12,13,14]. NPs accumulate in a tumor’s interstitium due to impaired tumor vasculature, a phenomenon known as the enhanced permeability and retention (EPR) effect [15], or passive targeting. Passive targeting can be enhanced using an active targeting approach, wherein ligands are implanted onto the NP’s surface, thus increasing the NPs uptake by cancer cells via receptor-mediated endocytosis [16,17]. The NP-based delivery of platinum agents has been previously explored [18,19], including liposome- [20,21], polymeric- [22,23], Bi_2_Te_3_- [24], or chitosan-based [25] NPs. Additionally, the use of biological [26] or inorganic carriers [27,28] have been reported. However, limitations to these systems exist, including low cellular uptake, uncontrollable drug release, or inadequate efficacy, each hampering the translational advancement of NPs to the clinic [29,30]. 

Recent developments in NP design seek stimuli-responsive “smart” building blocks that are sensitive to a tumor’s microenvironment, such as altered redox potential, enzyme upregulation, hypoxia, or acidic pH, to enhance the NP’s functions [31,32,33,34]. Some examples include pH-induced disruption of NPs for “on demand” drug release [35,36,37,38] or pH-promoted charge conversion to enhance the NPs uptake [39,40,41]. Additionally, chemical mediators, such as glutathione [42,43], as well as enzymes, such as metalloproteinases [44,45,46], hyaluronidase [47], or cathepsin B [48], have been proposed to trigger the disintegration of NPs and drug release.

Aptamers, also referred to as chemical antibodies, are short single-stranded nucleic acid sequences that fold into secondary or tertiary shapes and offer exceptional molecular recognition [49,50,51,52,53]. The target binding characteristics of aptamers and their small size, ease of chemical synthesis, the flexibility of chemical modification, and long-term storage stability prompted their use in place of conventional antibodies [50,54,55]. Still, bare oligonucleotides are prone to natural degradation by the nuclease enzymes present in the circulation and often exhibit short in vivo half-lives. Chemical modifications of the nucleobases have partially addressed these limitations, but these changes can distort the aptamers’ tertiary shapes and affect their target recognition characteristics [50].

One of the most common surfactants used to prolong the stability of NPs in serum is polyethylene glycol (PEG), as PEG-coated nanocarriers can evade the mononuclear phagocyte system (MPS) in the circulation [56,57]. PEG is a non-ionic hydrophilic polymer that provides a so-called “stealth” coating, diminishing the charge-based interactions of PEGylated nanostructures with plasma proteins [57]. However, studies have shown that the cellular uptake and endosomal escape of PEGylated NPs are suppressed, due to steric hindrance conferred by PEG [58,59,60]. Thus, PEGylated systems transformative in the tumor’s microenvironment are of great interest, and the nanoemulsions with metalloproteinase-sensitive PEG coating [61], solid-lipid NPs with pH-sensitive PEG for miRNA delivery [62], photothermal therapy [63], or pH-responsive micelles of PEG-siRNA [64] and PEG-acrylamide, as well as PEG-phosphatidylethanolamine conjugates [65,66], have been proposed to improve the NP’s translocation into the cells.

Herein, we report a pH-responsive PEGylated NP system with a poly(lactic-co-glycolic) acid (PLGA)-Pt (II) core for aptamer-based targeting of OC. We chose aptamer against Mucin1 [50] (MUC1), a glycoprotein present on the surface of normal epithelial cells, but overexpressed by at least 10-fold in OC cells [67]. The proposed pH-MUC1-Pt NP belongs to second-generation slow-releasing NPs, which are based on biocompatible and biodegradable PLGA [68]. PLGA exhibits a wide range of erosion times, has tunable mechanical properties, and importantly, is an FDA-approved polymer [69]. It has been extensively studied for the delivery of small molecules, proteins, and other macromolecules in commercial use and in research [70]. At present, many PLGA-based formulations are at the pre-clinical stage [70,71,72,73,74]. The rationale for the proposed NP system is to take the advantage of Pt (II) encapsulation to minimize the systemic toxicity of the drug, provide sustained release of Pt (II), and benefit from Apt-based targeting to increase NP’s specificity towards OC cells and increase the Pt (II) therapeutic index.

## 2. Material and Methods

More detailed schematic representation of synthetic methods is reported in Supporting Information.

### 2.1. Materials

Trifluoroacetic acid 99% (TFA), potassium carbonate 99%, silver nitrate, cis-dichlorodiamineplatinum (II) 99%, t-butyl-carbazide 97%, dicyclohexylcarbodiimide (DCC) were purchased from Acros Organics (Geel, Belgium); triethylamine, maleic anhydride 99% were purchased from Alfa Aesar (Ward Hill, MA, USA); polylactic-co glycolic acid (PLGA) was purchased from PolySciTech (West Lafayette, IN, USA), and poly(lactic-co-glycolic acid amine endcap (PLGA-NH_2_), cyanine7 N-hydroxysuccinimide mide ester (Cy7-NHS), and cyanine5.5 N-hydroxysuccinimide ester (C5.5-NHS) were purchased from Lumiprobe (Cockeysville, MD, USA); ELISA test (EHMUC1) and Slide-A-Lyser MINI-dialysis units were purchased from Thermo Scientific (Waltham, MA, USA); Bradford Assay was purchased from Bio-Rad (Hercules, CA, USA); 1,2-Distearoyl-sn-glycero-3-phosphoethanolamine (DSPE), 1,2-distearoyl-sn-glycero-3-phosphocholine (DSPC), 1,2-distearoyl-sn-glycero-3-phosphoethanolamine-N-[methoxy(polyethylene glycol)-2000] (ammonium salt) (DSPE-PEG2000), 1,2-distearoyl-sn-glycero-3-phosphoethanolamine-N-[methoxy(polyethylene glycol)-350] (ammonium salt) (DSPE-PEG350), 1,2-dipalmitoyl-sn-glycero-3-phosphoethanolamine-N-(glutaryl) (sodium salt) (DPPE-Glutaryl) were purchased from Avanti Lipids (Alabaster, AL, USA); O-[2-(6-Oxocaproylamino)ethyl]-O′-methylpolyethylene glycol 2000, MUC1 aptamer (custom ordered) were purchased from Sigma-Aldrich (St. Louis, MI, USA); DSPE-Thiol was purchased from Nanosoft Polymers (Winston-Salem, NC, USA); EDC-HCl was purchased from Oakwood Chemical (Estill, SC, USA). Fluorescein (FITC) was purchased from Fischer Scientific (Waltham, MA, USA).

All solvents were bought and used without further purification. Chloroform, ethyl acetate, methylene chloride (DCM), anhydrous ethyl ether, 2-propanol (IPA), N,N-dimethylformamide (DMF), methanol, hexanes were purchased from Fischer Scientific (ACS grade); acetonitrile HPLC grade; Alfa Aesar: anhydrous tetrahydrofuran (THF) and dimethyl sulfoxide (DMSO) were purchased from Acros Organics; ethyl alcohol 190 proof was purchased from DLI King of Prussia, PA, USA.

All experiments were performed in triplicates. Standard deviations, average values, *p*-values, and Z-test were performed using MS Excel (Microsoft Corporation, Redmond, WA, USA). 

### 2.2. Synthesis of pH-Sensitive DSPE-PEG

#### 2.2.1. Synthesis of 3-(2,5-Dioxo-2,5-dihydro-1H-pyrrol-1-yl)propanoic Acid (**1**)

First, 5.0 g (56.1 mmol) of beta-alanine was dissolved in 50 mL of glacial acetic acid, and then 6.6 g (67.3 mmol) of maleic acid anhydride was added portion-wise. The solution was stirred until the formation of a white precipitate, and the reaction mixture was subsequently refluxed overnight. Acetic acid was removed under reduced pressure at 40 °C. The remaining solid was suspended in 100 mL chloroform and filtered through a silica gel pad, followed by two additional washes with chloroform (2 × 100 mL). The filtrate was then condensed and dried under vacuum, affording 5.6 g (49% yield) of a white solid. ^1^HNMR (DMSO-d^6^) δ 12.35 (s,br,1H), δ 7.01(s,2H), δ 3.61 (t,2H), δ 2.51 (t,2H), ^13^CNMR (CDCl_3_) δ 172.06, 170.75, 134.61, 33.31, 32.41. HRMS-ESI: m/z [M + H]^+^ calc. for C_7_H_7_NO_4_: 170.0375; found: 170.0449 (Figure S1).

#### 2.2.2. Synthesis of Precursor tert-Butyl 2-(3-(2,5-dioxo-2,5-dihydro-1H-pyrrol-1-yl)propanoyl)hydrazine-1-carboxylate (**2**)

A total of 0.2000 g (1.18 mmol) of (**1**) and 0.2032 g (1.54 mmol) of t-butyl-carbazide was dissolved in 15 mL of DCM and chilled to 0 °C. The initial step was followed by the addition of 0.3172 g of DCC (1.54 mmol) in 5 mL of dichloromethane (DCM) to the reaction mixture. The reaction was allowed to warm up to room temperature. Reaction progress was monitored by TLC using a chloroform/methanol 4:1 solvent system. Upon completion (usually 1 to 2 h), the reaction mixture was chilled at −20 °C. The white precipitate was removed by filtration, and the filtrate was diluted with 30 mL of DCM and washed with 10% HCl solution (3 × 20 mL) and brine (20 mL). The organic phase was dried over anhydrous sodium sulfate and filtered. The crude product was purified by flash chromatography using hexane/ethyl acetate gradient, starting with 4:1 to 0:1 solvent ratio. The product was a colorless oil, 0.3829 g (87% yield). ^1^HNMR (CDCl_3_) δ 7.82 (s,br,1H), δ 6.70 (t,2H), δ 6.62 (s,br,1H) δ 3.86 (t,2H), δ 2.59 (t,2H), δ 1.44 (s,9H), ^13^CNMR (CDCl_3_) δ 172.43, 169.31, 155.34, 81.95, 33.81, 32.29, 28.08.

#### 2.2.3. Synthesis of 3-(2,5-Dioxo-2,5-dihydro-1H-pyrrol-1-yl)propanehydrazide (**3**)

A total of 0.2828 g (1.35 mmol) of (**2**) was dissolved in 10 mL of 20% solution of TFA in DCM and stored overnight. Next, the reaction mixture was condensed under reduced pressure and diluted with 3 mL of DCM, until the white product precipitated out. The product was chilled in the freezer at −20 °C for ~2 h and centrifuged at 4000 rpm (3148× *g*) and 4 °C for 5 min. The white solid was washed two times with 1.5 mL of cold DCM and then dried under vacuum. The product was a white solid, 0.1839 g (74.3% yield). ^1^H NMR (D_2_O) δ 6.87(s,2H), δ 3.84(t,2H), δ 2.65 (t,2H), ^13^C NMR (D_2_O) δ 172.56,171.38, 33.54, 31.98. HRMS-ESI: m/z [M + H]^+^ calc. for C_7_H_9_N_3_O_3_: 184.0644; found: 184.0710 (Figure S2, additional ^1^H NMR in CDCL_3_ Figure S5A and DMSO-*d6* Figure S5B)).

#### 2.2.4. Synthesis of Precursor (2,3-Bis(stearoyloxy)propyl (2-((1-(3-hydrazineyl-3-oxopropyl)-2,5-dioxopyrrolidin-3-yl)thio)ethyl) Phosphate (4)

A total of 0.0196 g (2.34 µmol) of DSPE-thiol was dissolved in 5 mL of dry chloroform, followed by addition of 0.0069 g (2.34 µmol) of (**3**) and 0.100 mL of triethylamine. The reaction mixture was allowed to stir at room temperature for 4 h. The progress of the reaction was monitored by NMR. Upon completion, the solvent and triethylamine were removed under reduced pressure. The crude product was dissolved in a minimum amount of chloroform and precipitated out with cold methanol. The product, 0.0180 g (75% yield), was a white solid. HRMS-ESI: m/z [M + H]^+^ calc. for C_51_H_95_N_4_O_12_PS: 1019.6405; found: 1019.6476 (Figure S3).

#### 2.2.5. Synthesis of Lipid-hydrazone-PEG2000 (**5**)

A total of 0.0150 g (1.47 µmol) of (**4**) was combined with 0.0294 g (1.47 µmol) of O-[2-(6-Oxocaproylamino)ethyl]-O′-methylpolyethylene glycol 2000 and stirred in 3 mL of chloroform. The progress of the reaction was monitored by NMR. Upon completion, the solvent was removed under vacuum. The product (**5**) was a white solid at 0.0350 g (78% yield). ^1^HNMR (CDCl_3_) (Figure S6).

### 2.3. The Synthesis of Phospholipid-MUC1 Conjugate

The MUC-1 aptamer-1,2-dipalmitoyl sn-glycero-3-phosphoethanolamine-N (DPPE)-Glutaryl conjugate was synthesized by standard EDC protocol. Briefly, MUC-1 5′amine-modified aptamer was dissolved in PBS buffer at pH 6.8. Next, at least 10× excess of DPPE-glutaryl was added, followed by the addition of EDC at a concentration of 0.2 M. The reaction was stirred overnight, then desalted using a QIAquick nucleotide removal kit. The final product was stored at −20 °C in a 20% ethanol/water solution. The final DNA concentration in the construct was analyzed by Nanodrop. 

### 2.4. Synthesis of PLGA-Pt (II) Conjugate

A total of 0.0652 g (217 μmol) of cisplatin was suspended in 5 mL of nanopure water and mixed with 0.0720 g (423 μmol) of silver nitrate in 1 mL of water. The reaction mixture was heated to 60 °C and stirred in the dark for 1 h. The white precipitate was filtered off, and the filtrate was added dropwise to 0.3000 g of PLGA (MW 1000–5000) dissolved in 15 mL of THF. The reaction was stirred at room temperature overnight. Next, the solvent was removed under reduced pressure, and the remaining solid was dissolved in acetonitrile. After centrifugation at 4000 rpm (3148× *g*) and 4 °C for 5 min, the supernatant was condensed and added dropwise to cold methanol. The pale brown precipitate was collected and dried under vacuum, producing 0.3020 g of the final product. The Pt (II) content was quantified by atomic absorption spectroscopy (AAS). 

### 2.5. Nanoparticle Synthesis

The pH-MUC1-Pt NP was synthesized via a nanoprecipitation method [75,76]. First, 5 mg of PLGA-Pt (II) conjugate was dissolved in 2.5 mL of acetonitrile to achieve a concentration of 2 mg/mL. Next, the PLGA-Pt (II) solution was added dropwise to the mixture of the following lipids: phospholipid-MUC1 (0.02 molar ratio to total lipids), 1 mg of DSPC/lipid-hydrazone-PEG (5) in (7:3 molar ratio) in 5 mL of 4% ethanol at 60–70 °C. The pH-MUC1-Pt NPs were allowed to stir for at least 2 h, and then the NP solution was washed three times with water using vivaspin filters (100,000 MW). The NP sample was condensed to achieve a volume of ~1 mL and characterized by transmission electron microscopy (FEI Titan Themis 200 kV, TEM, Thermo Fisher Scientific, Waltham, MA, USA) and dynamic light scattering (DLS). The Pt (II) content was analyzed by AAS. 

The synthesis of fluorescently labeled NPs, namely pH-MUC1-FITC and pH-MUC1-Cy5.5, followed the same protocol using PLGA-FITC and PLGA-Cy-5.5 conjugates, respectively.

### 2.6. Synthesis of PLGA-Cy5.5 Conjugate

A total of 101 mg (0.00363 mmol) of PLGA-NH_2_ (MW 28000) was dissolved in 2.5 mL of dry DMF and mixed with 2.6 mg (0.00363 mmol) of Cy5.5-NHS ester dissolved in 0.5 mL DMF. Next, 10 μL of triethylamine was added, and the reaction mixture was stirred overnight. After that, DMF was removed under reduced pressure, and the crude reaction mixture was dissolved in 1 mL of acetonitrile. The product was precipitated with cold methanol and stored at 4 °C, giving 49.7 mg (47% yield) of blue solid.

### 2.7. Synthesis of PLGA-FITC Conjugate

The synthesis was performed via click reaction between 49.3 mg (0.00159 mmol) of azide functionalized PLGA (MW 31,000) and 12 mg (0.0324 mmol) of propargyl fluorescein in the presence of 7.76 mg (0.00483 mmol) of copper sulfate and 19.98 mg (0.0113 mmol) of ascorbic acid in 6 mL of DMF at room temperature. Reaction mixture was stirred in the dark for 48 h. Next, the DMF was removed under reduced pressure. The semi-solid crude product was diluted with 5 mL of acetonitrile and centrifuged at 4000 rpm (3148× *g*) and 4 °C for 5 min to remove all solid impurities. The crude product was purified by dialysis in acetonitrile, using Spectrum Spectra/Por 6 pre-wetted standard RC Dialysis tubing 3500 MWCO, with four washes at 1, 4, 8, and 24 h. Purified product was condensed, affording 42 mg (84% yield) of pale-yellow solid. Product was stored in the dark at −20 °C.

### 2.8. Synthesis of DSPE-Cy7 Conjugate

A total of 13.7 mg (0.0183 mmol) of DSPE was dissolved in 1 mL of dry DMF. Next, 12.5 mg (0.0183 mmol) of Cy7-NHS ester in 0.5 mL of DMF was added to the solution, followed by 10 μL of triethylamine, and the reaction mixture was stirred overnight. After that, DMF was removed under reduced pressure. The product was washed 3 times with cold acetonitrile and then dissolved in ethanol, giving 18 mg (77% yield) of blue solid. DSPE-Cy7 conjugate labeled was added to the NP’s coating at 5 mol.% to facilitate in vivo imaging.

### 2.9. Characterization

The pH-MUC1-Pt NPs’ size and zeta potential were analyzed by dynamic light scattering (DLS) and ZetaPals (Brookhaven Instrument Corporation). The concentrated NPs solution was diluted in distilled water to obtain a concentration of 0.500 mg PLGA/mL. The obtained solution was measured for the hydrodynamic mean diameter and size distribution. Each measurement was performed in triplicate. The shape, surface morphology, and size of the NPs were analyzed by TEM. The NP samples were mixed with the acetate buffer (0.125 M CH_3_COONH_4_, 0.6 mM (NH_4_)_2_CO_3_ and 0.26 mM tetrasodium EDTA at pH 7.4). A total of 10 μL of the sample was negatively stained with 10 μL of 2% (*w*/*v*) phosphotungstic acid. A droplet of the NPs was placed on a carbon-coated copper grid (Electron Microscopy Sciences, CF400-Cu, carbon film, 400 mesh), forming a thin liquid film.

### 2.10. In Vitro Drug Release

The in vitro Pt (II) release studies from pH-MUC1-Pt NPs were carried out using a dialysis bag diffusion method [67]. In brief, 500 μL of the NP solution was dispensed into mini-dialysis tubes (Slide-A-Lyser MINI-dialysis units, Thermo Scientific), and the tubes were placed into three separate beakers containing 600 mL of pH 7.4, 6.8, and 5.5 phosphate buffer, respectively. A total of 600 mL of buffer was used to create dialysis/osmosis gradient for sufficient platinum transfer. The samples were gently stirred. The buffer temperature was maintained at 37 ± 1 °C throughout the experiment. Samples (three tubes) were withdrawn at defined time intervals and analyzed for Pt (II) concentration with AAS. Drug release = D_t_/D_0_ × 100. From the above formulae, D_t_ and D_0_ indicate the amount of drug released from the NPs at certain intervals and the total amount of drug in the NPs solution, respectively. D_t_ = (D_0_ – Remaining amount of Pt in the tube).

### 2.11. Fluorescamine Test

The pH-MUC1 NP solution was diluted with three solutions of PBS buffer at pH 7.4, 6.8, and 5.5, respectively, to achieve a final concentration of 1.6 mg pH-MUC1 NP/mL. Next, 10 μL of fluorescamine solution in DMSO (3 mg/mL) was mixed with 150 μL of each NP solution. Each solution was then transferred into a black flat bottom 96-well plate and analyzed using a plate reader (Spectra Max M3 by Molecular devices). PBS solutions at pH 7.4, 6.8, and 5.5 and PBS at pH 7.4, 6.8, and 5.5 with pH-MUC1 NPs without fluorescamine were used as controls. 

### 2.12. Cellular Uptake of the NPs

For flow cytometry experiments comparing the NPs uptake between pH 7.4 and 6.8, A2780 and CP70 cells were seeded in 6-well plates (at a density of 1 × 10^5^ cells/well) and treated with 600 μg/mL of pH-MUC1-FITC NPs at pH 7.4, pH-MUC1-FITC NPs preincubated for 1 h in cell media at pH 6.8, and pH-MUC1-FITC NPs with PEG 350 for 15 min at 37 °C. Next, the cell culture medium containing the NPs was removed, and the cells were washed twice with PBS, digested by trypsin, and harvested by centrifugation at 1250 rpm (307× *g*) for 5 min. Finally, the fluorescence intensity of FITC was detected using a flow cytometer (FACS Calibur BD Biosciences) at an excitation wavelength of 488 nm and an emission wavelength of 530 nm.

For real-time uptake experiments, CP70 cells were plated in a four-well round chamber (Greiner Bio-one) at a density of 5 × 10^4^ cells/well. After 24 h, wheat germ agglutinin (WGA) Alexa Fluor^TM^488 conjugate (Invitrogen) was added to the media at a concentration of 5 μg/mL. Next, the pH-MUC1-Cy5.5 NPs were added to the cells. The pH of the media was 6.8. Imaging started 5 min after the addition of the NPs and continued for 10 min. Imaging was performed using an LSM880 inverted confocal microscope (Zeiss, Jena, Germany) with Airyscan and FAST.

### 2.13. ELISA Test for Mucin 1 Presence in OC Cells

Muc1 test was purchased from Thermo Scientific (EHMUC1). A total of 1 × 10^7^ cells of each type were harvested and washed 3x with PBS. Next, the cells were suspended in 400 μL of a lysis buffer made of 50 mM Tris buffer, 100 mM NaCl, and x1 of protease inhibitor cocktail (Thermo-Scientific cat.#7448). Protein extraction was performed by sonication using the QSONICA Q500 sonicator. Six five-second sonications were followed by a 30 s cooldown time. Sonication was performed on ice. Protein extract was centrifuged at 15,000 rpm (21,130× *g*) for 30 min at 4 °C. The supernatant was collected and stored on ice. Protein content was tested using the Bradford protocol. ELISA test was performed according to the manufacturer’s guidelines.

### 2.14. In Vitro Viability Assay

Ovarian Cancer cell lines A2780, CP70, OVCAR-3, SKOV-3, OV-90, TOV-21G, ES-2 were purchased from American Type Culture Collection (ATCC, Manassas, VA, USA). Cell lines were cultured in Dulbecco’s modified eagle medium (Sigma Aldrich) supplemented with 10% (A2780, CP70, SKOV-3, ES-2) or with 15% (OVCAR-3, TOV-21G, OV-90) fetal bovine serum (FBS, HyClone) with L-glutamine (HyClone) and penicillin/streptomycin (HyClone). All cells were grown in a water-saturated atmosphere at 37 °C and 5% CO_2_. Cells were seeded at a density of 3 × 10^5^ per well in 96-well plates and pre-cultured overnight. The pH-MUC1-Pt NPs were added at the concentrations corresponding to IC_50_ for each cell line and incubated for 72 h. After 72 h, media was aspirated, and cells were washed with PBS. Then, a 10% Alamar Blue solution in PBS was added to each well, and the cells were incubated for an additional 30 to 45 min. The metabolic activity of the cells was determined by measuring fluorescence at 590 nm (plate reader Spectra Max M3 by Molecular Devices). 

### 2.15. DNA:Pt Quantification

Cells were seeded onto Petri dish at the density of 3 × 10^5^ cells/Petri dish and cultured as in 2.14. The pH-MUC1-Pt NPs were added at the concentrations corresponding to IC_50_ for each cell line and incubated 72 h. After 72 h, media was aspirated, and cells were washed with PBS. Cells were treated with trypsin for approximately two minutes, followed by the addition of the media. After centrifugation at 1250 rpm (307× *g*) for 10 min, cells were washed with PBS three times and centrifuged again. Whole cellular DNA was isolated using QIAamp^®^ DSP DNA Mini Kit (QIAGEN, Hilden, Germany), according to the manufacturer instructions. The concentration of DNA was measured using NanoDrop 2000 C Spectrophotometer (ThermoScientific). Aliquots from the DNA samples were lyophilized and treated with 50 μL of aqua regia. After 24 h, 450 μL of nanopure water was added, and the samples were analyzed for platinum content using AAS. The same protocol was used in the experiment where Pt:DNA ratio was measured over time, the A2780 cells were harvested after 6, 24, and 72 h at pH 6.8 and 7.4.

### 2.16. In Vivo Mouse Model Studies

All in vivo experiments were approved by the Institutional Animal Care and Use Committee (IACUC) of the Icahn School of Medicine at Mount Sinai. Five-week-old female nude mice (Charles River, MA, USA), weighing 18–20 g and maintained in specific pathogen-free conditions, were used to generate the OC tumor model and to determine the in vivo half-life of the NPs. A total of 1 × 10^7^ OVCAR-3 cells were inoculated subcutaneously into the dorsal region near the hind limb of nude mice (n = 3). When resultant tumors reached approximately 500 mm^3^, as measured using external calipers, mice were treated with pH-MUC1-Pt-Cy7 NPs via tail vein injection at a concentration of 0.314 mg of Pt (II). A total of 24 h after the injection, in vivo fluorescence imaging was performed as described below. Mice were then euthanized, and subcutaneous tumors and organs (brain, lung, liver, kidney, spleen, and heart) were collected for ex vivo fluorescence imaging and AAS analysis. 

### 2.17. In Vivo Fluorescence Imaging

In vivo and ex vivo, near-infrared fluorescence (NIRF) imaging experiments were performed using the IVIS-200 System (Xenogen, Alameda, CA, USA). To enable detection of the Cy7 labeled NPs, 745 nm excitation and 800 nm emission filters were used. A field of view (FOV) of 17.6 and an excitation time of 4 s were chosen.

### 2.18. AAS Analysis of Pt (II) in Organs

Excised organs were frozen, lyophilized, and dissolved in aqua regia for 48 h. Next, the samples were sonicated for 24 h in a water-bath sonicator and dried under vacuum. After that, 200 μL of water was added to each sample, all samples were centrifuged at 15,000 rpm (21,130× *g*) and 4 °C for 30 min, and the supernatants were directly analyzed by AAS.

## 3. Results and Discussion

**Figure 1:**
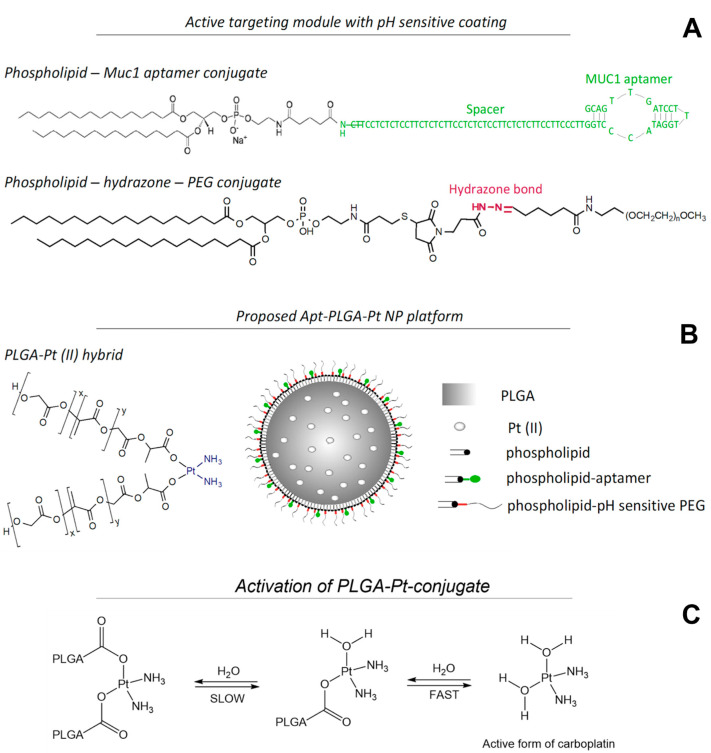
A schematic of an active targeting module (**A**), the proposed pH-MUC1-Pt NP platform (**B**), and activation of Pt complex from PLGA-Pt (II) hybrid (**C**).

The designs of pH-sensitive PEG and phospholipid-MUC1, as well as pH-MUC1-Pt NP and Pt (II) activation in the PLGA-Pt hybrid, are schematically presented in Figure 1 [77]. The coating of the NP includes a pH-sensitive phospholipid-hydrazone-PEG conjugate that was formed from PEG2000-aldehyde and 1,2-Distearoyl-sn-glycero-3-phosphorylethanolamine (DSPE)-phospholipid-hydrazine precursors (Figure S7). The reaction was monitored by ^1^H NMR, where the successful disappearance of the CHO aldehyde signal at 9.8 ppm was indicative of the formation of hydrazone bond between PEG-aldehyde and phospholipid-hydrazine (Figure S8 and full spectra Figure S4). The targeting part of the NP’s coating involves a phospholipid-MUC1 conjugate. The synthesis of the conjugate followed a standard EDC coupling chemistry between an amine-terminated MUC1 and a carboxylate-terminated 1,2-dipalmitoyl-sn-glycero-3-phosphorylethanolamine (DPPE) phospholipid. The final DNA content after the synthesis was quantified using a UV–Vis spectrophotometry and was determined to be 46.7% by mass, corresponding to an ~1:31 molar ratio of DNA Apt to DPPE phospholipid. 

The core of the NP consists of PLGA polymer chemically functionalized with Pt (II). The PLGA-Pt (II) hybrid was formed by the method developed previously by our group for Pt (II)-lipophilic carboxylates [78]. The coordination of PLGA to the Pt (II) center in the hybrid resembles a configuration of carboplatin and is expected to follow the same mechanism of action. This involves activation of Pt (II) complex by acid hydrolysis with a successive displacement of two monodentate carboxylates, initiated with a ring-opening step, followed by a complete loss of ligand [79,80]. The process is pH-sensitive, and, at high acidity, the first step is ten times faster than the second, while at low acidity, this difference is four-fold [81]. The final active species is cis-[Pt(NH_3_)_2_(H_2_O)_2_]^2+^, which enters the nucleus via electrostatic interactions with negatively-charged DNA facilitating the formation of DNA adducts [1]. The PLGA-Pt (II) hybrid was characterized with atomic absorption spectroscopy (AAS) and revealed the Pt (II) concentration of 3.62% by mass, which corresponds to 1:1.7 molar ratio of Pt (II) to PLGA, assuming the average molecular weight of PLGA to be 3000 Da. This ratio suggests the coordination of two PLGA monomers to one Pt (II) center. 

**Figure 2:**
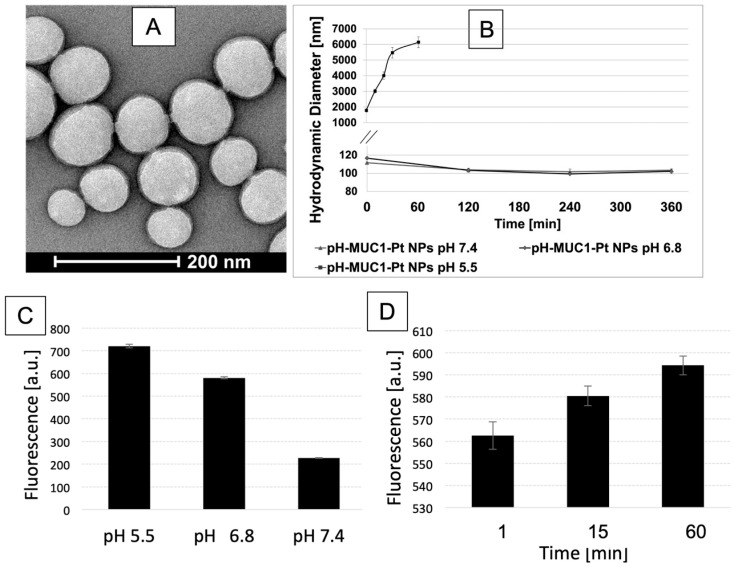
(**A**) TEM image of negatively stained pH-MUC1-Pt NPs; (**B**) stability of pH-MUC1-Pt NPs in 10% FBS at pH 7.4, 6.8 and 5.5; (**C**) Measurement of the hydrazone bond cleavage of the pH-MUC1 NP via fluorescamine test after incubation of the NP at different pHs; (**D**) Time dependent hydrazone bond cleavage of pH-MUC1 NP at pH 6.8. Data in (**B**–**D**) presented as mean ± st. dev (*n* = 3).

The pH-MUC1-Pt NPs were characterized with respect to their size, surface charge, stability, and in vitro Pt (II) release. The size of the NPs was examined by dynamic light scattering (DLS) and transmission electron microscopy (TEM). The hydrodynamic diameter of the NPs studied by DLS was found to be 110 nm, with a low polydispersity of 0.199. The Zeta potential of the NPs was determined to be −29 mV, indicating good colloidal stability and a negative surface charge, where the latter is expected to prevent the non-specific NP–protein adsorption in vivo that might lead to cytotoxicity [32]. The TEM image of pH-MUC1-Pt NPs is shown in Figure 2A and reveals that the NPs have a spherical morphology, with a core diameter of 92 ± 15 nm. This size range is suitable for in vivo applications of the NPs and reaching the tumor site by crossing the barrier of leaky tumor vasculature. The openings in the defective endothelium range from 200 nm to 1.2 µm, and NPs with diameters below this size effectively penetrate the vessel walls [82,83,84].

Next, we evaluated the stability of pH-MUC1-Pt NPs in fetal bovine serum (FBS) at pH 7.4, 6.8, and 5.5 to determine if PEG coating prevents the opsonization of the NPs with plasma proteins and if it protects the DNA aptamer from enzymatic degradation. FBS is a blood product that contains a variety of active proteins and nucleases [85,86], most importantly, DNase I that can lead to the digestion of nucleic acids [87]. In the experiment, the pH-MUC1-Pt NPs were mixed with 10% FBS solution at a final NPs concentration of 600 µg/mL and incubated at 37 °C for 6 h. The size of the NPs was measured by DLS at different time points. The results are shown in Figure 2B. The diameter of the pH-MUC1-Pt NPs remained stable during the test period at pH 7.4 and 6.8, suggesting the absence of any NP–NP and NP–protein aggregates, which would include the nucleases. Therefore, the NPs could be expected to be stable in the systemic circulation and upon entry to the tumor. However, further lowering the pH to 5.5 resulted in the quick increase of the NPs hydrodynamic diameter, suggesting complete cleavage of pH-PEG and immediate aggregation of the NPs. Since the pH of 5.5–7.0 [88,89] is common for diseased tissues, such as tumors, the NPs are expected to completely shed pH-PEG in the tumor’s microenvironment, facilitating the active targeting of cancer cells via DNA aptamers.

The direct measurement of the hydrazone-PEG bond cleavage on the pH-MUC1 NP (no Pt) was monitored using a fluorescamine test. The hydrolysis of hydrazone bond yields a free amine, and a non-fluorescent fluorescamine in the presence of free amine produces a highly fluorescent product. To this end, the pH-MUC1 NP solutions were incubated with fluorescamine for 15 min at pH 7.4, 6.8, and 5.5 in black 96-well plates. The result of the test is presented in Figure 2C. It can be clearly seen that the fluorescence is higher at pH 6.8 versus pH 7.4 and the highest at pH 5.5, indicating a faster hydrazone bond hydrolysis of the pH-sensitive PEG with decreasing the pH. Additionally, we monitored the fluorescence change over time (60 min) at tumoral pH of 6.8 using the same test (Figure 2D). The fluorescence increases with longer incubation times, confirming the continuous hydrazone-PEG bond cleavage and formation of free amines in a low acidity environment. Efficient PEG shedding in the tumor microenvironment is needed to facilitate interactions between NPs and cancer cells. During in vivo delivery of the NPs, the cellular membrane provides another challenge to cellular internalization of the NPs [90]. Active targeting relies on effective ligand–receptor binding for improved accumulation of the NPs in the cancer cell [84]. Therefore, complete exposure of the targeting ligands, upon PEG cleavage, in the cancer cell vicinity is of utmost importance for essential ligand–receptor interactions and effective NPs uptake into the cells. 

**Figure 3:**
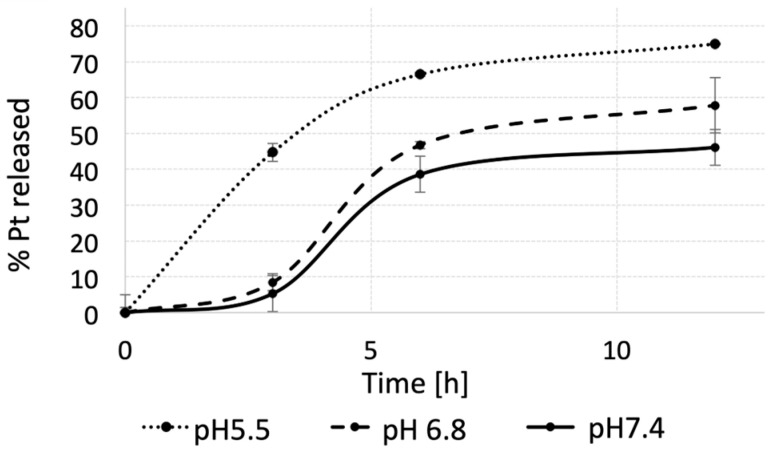
In vitro Pt (II) release from pH-MUC1-Pt NP in PBS at 37 °C. Data presented as mean ± st. dev. (*n* = 3).

The in vitro Pt (II) release from the pH-MUC1-Pt NPs was studied at three different pH values: 7.4 (physiological), 6.8 (tumor’s interstitium), and 5.5 (tumoral, endosomal). The degradation rate of the PLGA polymer is pH-dependent [91], which can influence Pt (II) release from the NPs. The initial Pt (II) payload in the pH-MUC1-Pt NPs was determined by AAS and was found to be 1.62 wt.%. The NP solutions at the concentration of 600 µg/mL were placed in mini-dialysis tubes and incubated at 37 °C in PBS for 12 h. The solutions were collected from the dialysis tubes at pre-defined time intervals and quantified for Pt (II) content using AAS. The percentage of Pt (II) released, with respect to the initial Pt (II) concentration, is presented in Figure 3. After 3 h, only 2.5% of Pt (II) was released at pH 7.4, 9% at pH 6.8, and 45% at pH 5.5. The Pt (II) release from the NPs after 12 h at pH 7.4 was lower by 14%, when compared to pH 6.8 and by 30% versus pH 5.5, showing a consistent trend with the earlier time point. This result suggests that the Pt (II) release would be low in the circulation after the intravenous administration of the NPs and heightened once the NPs translocate into the tumor space, as well as into intracellular compartments. In addition, and based on these experiments, the pH-MUC1-Pt NP follows the characteristics of a slow-releasing system, as expected for PLGA-based NPs, which is of high importance in the drug delivery field [68].

**Figure 4:**
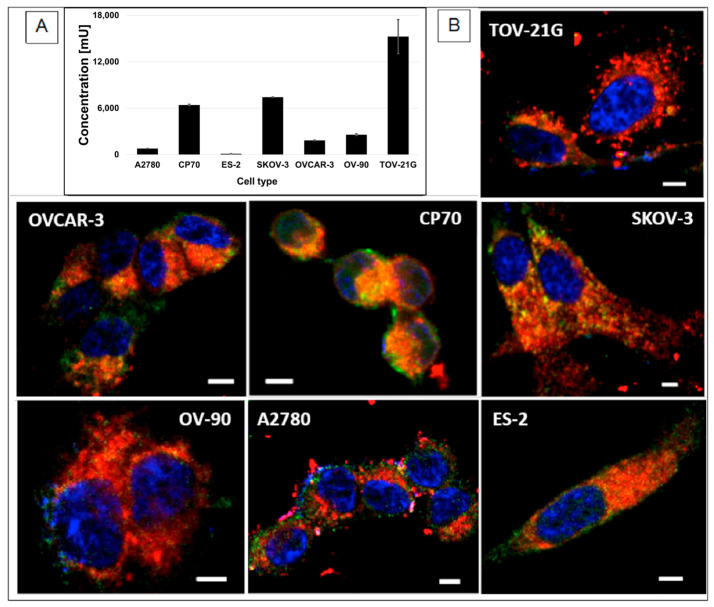
(**A**) ELISA test results for MUC 1 expression in OC cells. Data in presented as mean ± st. dev (*n* = 3). (**B**) Confocal microscope images of OC cell lines after 15 min incubation with pH-MUC1-Cy5.5 NPs. The NPs are shown in red, MUC1in green, and nuclei are in blue. Scale bars are 5 µm in all images.

Epithelial ovarian cancer (EOC) is the most common type of cancer and accounts for about 90% of all ovarian cancers. MUC1 is a transmembrane glycoprotein that is overexpressed in many types of cancer, including EOC [92]. MUC1 is a major component of the mucin layer that lines the epithelial cells. In EOC, MUC1 is associated with poor prognosis, tumorogenicity, and metastasis and progression. Therefore, MUC1 is an attractive cancer cell receptor in OC therapy [93]. To validate the targeting strategy, we evaluated MUC 1 expression using an ELISA test in different OC cells. We used the isogenic A2780 (Pt-sensitive) and CP70 (Pt-resistant) cell lines [94], as well as cells with different genomic features, histotypes, and known Pt (II) resistance characteristics [95]. The results are presented in Figure 4A. MUC1 expression is present in all cells tested with the lowest in ES-2 and the highest levels in TOV-21G, SKOV-3, and CP70 cells. 

Subsequently, we analyzed NP uptake by all OC cell lines with confocal microscopy. We used fluorescent pH-MUC1-Cy5.5 NP (no drug), and we tested the propensity of the NPs to associate with expressed MUC1 (Figure 4B). To this end, cells were incubated with the NPs for 15 min, fixed and incubated with primary murine anti-human MUC1 antibody, followed by secondary goat anti-mouse antibody labeled with Alexa 488. The NPs (red) are seen to be associated with MUC1 (green) and are homogeneously distributed within the OC cells. Most importantly, NP association with MUC1 appears strong even intracellularly, suggesting that these NPs may exploit MUC1’s intracellular pathway. This result further validates the use of MUC1 as a target for efficient NPs delivery into the cytoplasm. Importantly, the NPs can penetrate the cell membrane and are abundant inside the cell, which is necessary for effective drug delivery into the cancer cells.

**Figure 5:**
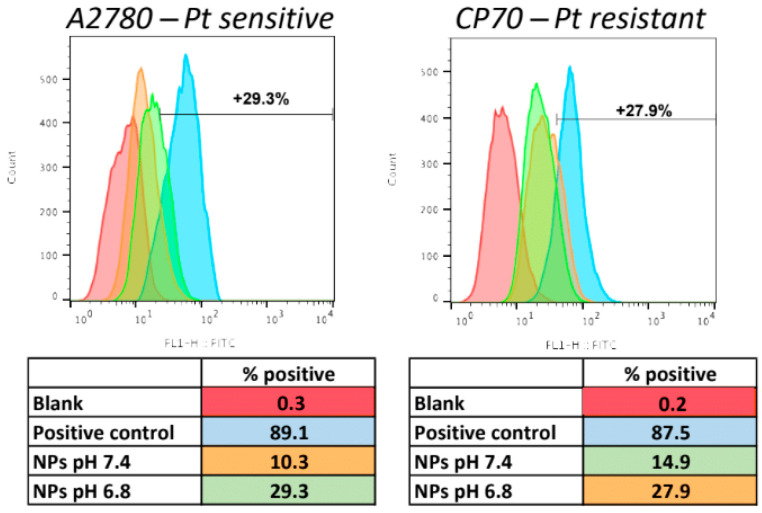
Flow cytometry analysis of A2780 and CP70 cells after 15 min incubation with pH-MUC1-FITC NP.

Next, we performed a pilot experiment to observe if there are detectable differences in NP uptake at different pH levels. We, therefore, incubated the two isogenic cell lines A2780 and CP70 with fluorescent pH-MUC1-FITC NPs for 15 min, at either pH 7.4 or 6.8. We used MUC1-FITC NPs coated with PEG 350, which preserves surface exposed MUC1 as a positive control, and compared the results to cells only (Figure 5). The results are summarized in the tables below the histograms. Even after only a short incubation period of 15 min, there was a demonstrable increased uptake of the NPs at pH 6.8 in both cell lines, with ~3- and 2-times higher uptake in A2780 and CP70 cell lines, respectively. This result suggests increased interactions of the NPs with the cells at lower pH, which originates from quicker PEG shedding and enhanced exposure of the targeting ligands on the NP’s surface. This increased NP-cell interaction at lower pH leads to higher uptake of the NPs via receptor-mediated endocytosis. 

To corroborate these findings, we performed real-time confocal imaging of NP entry into the cells at pH 6.8. To this end, the CP70 cells were cultured in confocal microscope-ready Petri dishes, and the pH of the cell media was adjusted to 6.8 before the addition of the NPs. The cellular membrane was stained with wheat germ agglutinin (green). The recording started 5 min after the addition of pH-MUC1-Cy5.5 NPs to the cells and continued for 10 min. A representative time frame (24 s) is presented in Supplementary Information. Cellular uptake of the NPs was apparent (red fluorescence) and consistently increased over time, with the NPs appearing to be localized within the cells. Since cytoplasmic localization of the NPs is a necessary precondition for Pt (II) release and activity, these results suggest that the NP can be used as an efficient nanocarrier for the intracellular delivery of Pt (II). 

**Figure 6:**
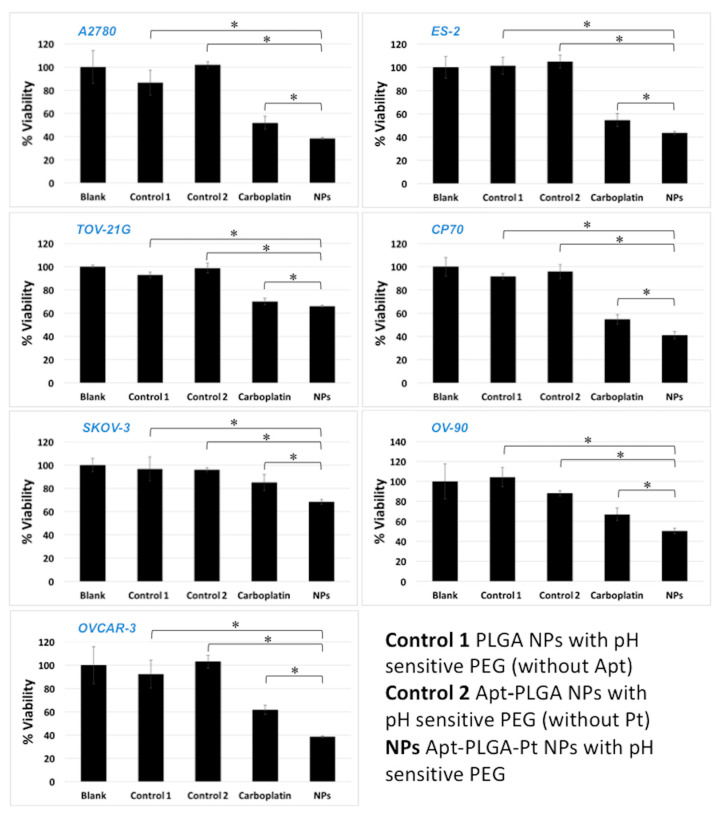
Cellular viability of seven different OC cell lines, following 72 h incubation with pH-MUC1-Pt NP and controls. Data presented as mean ± st. dev (*n* = 3) and *p* < 0.01 all cell lines, *p* < 0.2 in TOV-21G. Asterisks denote a statistically significant difference. The concentrations of Pt(II) are constant in each cell line and are as follows: SKOV-3 3.44 × 10^−5^ M, CP70 5.28 × 10^−5^ M, OV-90 2.95 × 10^−5^ M, TOV-21G 2.95 × 10^−5^ M, A2780 3.08 × 10^−5^ M, OVCAR-3 3.08 × 10^−5^ M, ES-2 3.44 × 10^−5^ M.

We have also determined the IC_50_ values and performed viability assays (Alamar Blue) in the OC cell lines using predetermined IC_50_ values. As shown in Figure 6, we compared the pH-MUC1-Pt NPs to cells only, empty NPs: pH NPs (without MUC1 and Pt) and pH-MUC1 NPs (without Pt), as well as free carboplatin. For all experiments, cell lines were seeded in 96-well plates and incubated with pH-MUC1-Pt NPs for 72 h at a Pt (II) concentration, corresponding to the experimentally determined IC_50_ values obtained for the NPs (Figures S9–S15). Cell viability using control groups (empty NPs) were similar to the untreated cells. Viability was markedly decreased in the pH-MUC1-Pt NPs and free carboplatin groups, respectively, by 61.0% and 49.1% (A2780), 55.5% and 43.5% (ES-2), 34.2% and 31% (TOV-21G) in Pt-sensitive cell lines, when compared to untreated control groups after 72 h of incubation. In Pt-resistant cells, the same trend was observed with viability reduction of 59.7% and 42.7% (CP70), 50.4% and 32.0% (OV-90), 50.3% and 12.2% (SKOV-3), and 61.5% and 39.3% (OVCAR-3) for pH-MUC1-Pt NPs and free carboplatin groups, respectively. In all cases, the biological activity of the pH-MUC1-Pt-NP formulation was higher than that of the free drug. 

**Figure 7:**
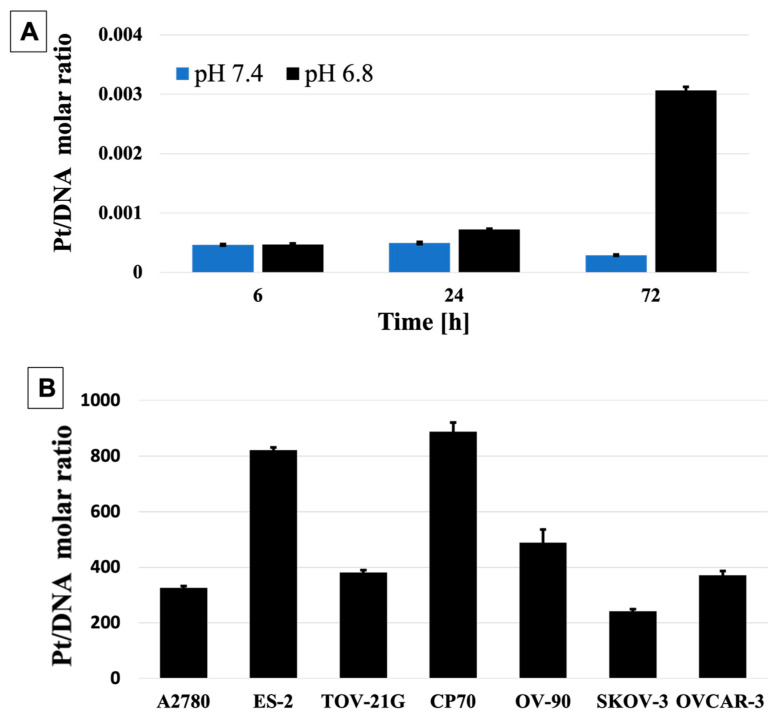
(**A**) Pt:DNA molar ratio in A2780 cells after incubation with pH-MUC1-Pt NP. (**B**) The ratio of Pt (II) to DNA base pairs in all OC cell lines following 72 h incubation with pH-MUC1-Pt NP. Data in (**A**,**B**) presented as mean ± st. dev (*n* = 3). The concentrations of Pt (II) in all cell lines are the same as used for in vitro studies.

In addition, we calculated the exact platinum-to-DNA base pair ratio for all cell lines tested, using a method previously developed by our group [96]. Shortly after incubation with the NP, cells were trypsinized and lysed, and the DNA was recovered and quantified using nanodrop. Pt (II) concentration in the same DNA samples was established using AAS. Initially, we incubated A2780 cells with pH-MUC1-Pt NP for 6, 24, and 72 h to examine the time dependent concentration of Pt (II) in the nucleus. The results are presented in Figure 7A and show that the amount of Pt (II) bound to DNA increases over time, indicating continuous Pt (II) delivery into the nucleus. Next, we evaluated the Pt (II) in the nuclei of all OC cells after 72 h of incubation with the NP (Figure 7B). The Pt:DNA ratios were as follows: 1:326 (A2780), 1:822 (ES-2), 1:381 (TOV21-G), in Pt-sensitive cell lines, and 1:888 (CP70), 1:489 (OV-90), 1:242 (SKOV-3), 1:371 (OVCAR-3) in Pt-resistant cell lines. Since there are ~10 base pairs per turn in a helix, this gives one Pt (II) attached every 31st turn in A2780, 78th in ES-2, and 36th in TOV-21G cells, as well as every 85th in CP70, 47th in OV-90, 23rd in SKOV-3, and 35th turn in OVCAR-3 cells. Together, these results indicate that the pH-MUC1-Pt NP is a slow releasing system, capable of effective Pt (II) delivery to DNA, and it has therapeutic potential towards OC cells.

To examine the pharmacokinetic properties of the pH-MUC1-Pt NP, we used fluorescence and AAS to measure NPs concentration in blood samples collected at various time points following their administration in mice. Lipids labeled with a near-infrared (NIR) Cy7 fluorophore were added to the NP’s coating at 5 mol.% to facilitate in vivo imaging. The pH-MUC1-Pt NPs were administered intravenously to nude mice at 0.314 mg Pt (II). The dose given to mice corresponded to a bioequivalent cisplatin dose for patients (75 mg/m^2^) (Figure S16) [97]. Blood samples were drawn at 5, 20, 45, and 120 min, where the two-hour range of the sampling was chosen arbitrarily based on the drug release study (Figure 3). At physiological pH, the drug release is below 10% within the 2-h range. Blood was centrifuged, and the plasma was collected for analysis. The blood plasma was added to a 96-well clear bottom plate and fluorescence was recorded using a plate reader. Additionally, the blood samples were processed for AAS analysis, as described in the Materials and Methods section. Exponential decay analysis of pH-MUC1-Pt NP revealed the in vivo half-life of 45 min. 

**Figure 8:**
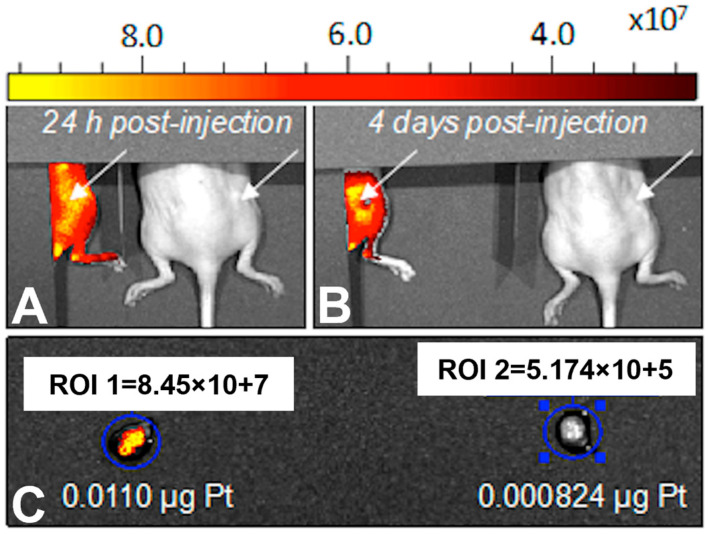
The images of NPs biodistribution in vivo (**A**,**B**) in an ovarian cancer mouse model and excised tumors (**C**) from an NP-injected mouse (**A**) and a control mouse (**B**). White arrows indicate the tumor site. The data were collected with the help of the “Small Animal Imaging Center in the Translational and Molecular Imaging Institute.”

Next, we evaluated the potential of pH-MUC1-Pt NP to target solid tumors in a pre-clinical model of ovarian cancer. The pH-MUC1-Pt NPs, at the same Pt (II) dose as in half-life studies, were intravenously injected via tail vein into OVCAR-3 tumor-bearing nude mice. The NP biodistribution studies in vivo were performed using Cy7-labeled NPs and an IVIS small animal imaging system. Untreated (saline only) mice were used as a control. A total of twenty-four hours post-injection, no fluorescence signal was detected in the control mouse, and a strong NIR signal was observed at the tumor site in the pH-MUC1-Pt NP injected mice (Figure 8A). In addition, after four days, the NIR signal in the tumor was still high, indicating a prolonged retention of the NPs in the tumor (Figure 8B).

After four days, the tumors and organs were excised and examined ex vivo by fluorescence and AAS. Figure 8C demonstrates fluorescence in the excised tumor, as measured by regions of interest (ROIs). The AAS measurements of Pt (II) concentration reveals the presence of 0.011 µg of Pt in the extracted tumor with the NPs and a negligible Pt amount in the control. Collectively, these results strongly indicate that the NPs effectively translocate into tumors and are retained in the tumor’s interstitium for several days. Moreover, the pH-MUC1-Pt NPs deliver Pt (II) directly to cancer cells in vivo. 

**Figure 9:**
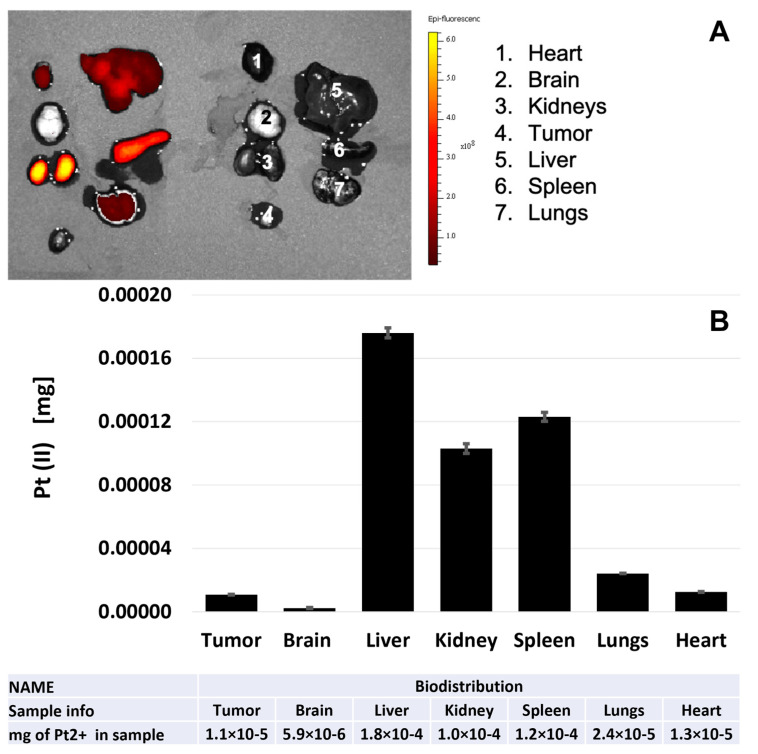
(**A**) Ex-vivo study of Apt-PLGA-Pt NPs biodistribution in organs at four days after the NPs injection (left) and control untreated organs (right). The data were collected with the help of the “Small Animal Imaging Center in the Translational and Molecular Imaging Institute.” (**B**) AAS analysis of a platinum (II) biodistribution in organs at four days after the NPs injection.

The background biodistribution of the NP into the organs was also assessed, and the NIR images of the organs, as well as AAS quantification of Pt (II), are presented in Figure 9. The NPs were detected in the liver, spleen, and kidneys and in much lesser amounts in the heart and in the lungs. No signal was detected in the brain, indicating that the pH-MUC1-Pt NPs do not cross the blood–brain barrier. The mononuclear phagocyte system (MPS), composed of macrophages monocytes and dendritic cells, is involved in the sequestering of the NPs from the bloodstream leading to NPs accumulation in the liver and spleen [32]. However, a high content of the NPs was also detected in the heart, lungs, and kidneys. All these organs are characterized with high blood perfusion, which could contribute to accumulation of the NPs in these organs directly from the blood compartment. Therefore, the additional accumulation of the NPs in the tumor site over time could be possible via the circulatory system. 

## 4. Conclusions

In summary, a novel pH-sensitive, NP-based system for active targeting and effective Pt (II) delivery to tumors was synthesized and characterized. The pH-MUC1-Pt NP demonstrated cellular uptake across various OC cell lines, resulting in in vitro efficacy, especially in cell lines known to be Pt-resistant. Importantly, in vivo studies confirmed the suitability of the platform for tumor targeting and drug delivery, as the NP effectively translocated to the tumor and Pt (II) was detected in the excised cancer cells, respectively. Taken together, we believe these findings suggest that the pH-MUC1-Pt NP platform can be used for Pt (II) delivery. Future pre-clinical survival studies using mouse models are planned to test the efficacy of the platform. Finally, and with the possibility of improving targeting and widening the scope of cancers treated, the modular design of the NP allows for quick modifications, and the platform could easily be extended to other polymeric or lipophilic NP-based systems.

## Data Availability

All the data generated within this study were provided in supplementary materials.

## Supplementary Materials

The following supporting information can be downloaded at: https://www.mdpi.com/article/10.3390/pharmaceutics15020607/s1, Figure S1: HRMS of (3-(2,5-dioxo-2,5-dihydro-1H-pyrrol-1-yl)propanoic acid) (**1**); Figure S2: HRMS of (3-(2,5-dioxo-2,5-dihydro-1H-pyrrol-1-yl) propanehydrazide) (**3**); Figure S3: HRMS of (2,3-bis(stearoyloxy)propyl (2-((1-(3-hydrazineyl-3-oxopropyl)-2,5-dioxopyrrolidin-3-yl)thio)ethyl) phosphate) (**4**); Figure S4: ^1^H-NMR spectrum of: Top to bottom: DSPE-Thiol, 3-(2,5-dioxo-2,5-dihydro-1H-pyrrol-1-yl)propanehydrazide (**3**), PEG-2000 aldehyde and lipid-hydrazone-PEG2000 (**5**); Figure S5: ^1^H-NMR spectrum of: 3-(2,5-dioxo-2,5-dihydro-1H-pyrrol-1-yl)propanehydrazide (**3**) (A) in CDCl_3_; (B) in DMSO-*d6*; Figure S6: ^1^H-NMR spectrum of: Lipid-Hydrazone-PEG2000 (**5**) in CDCl_3_; Figure S7: Schematic of the synthetic approach to pH-sensitive PEG2000-hydrazone-phospholipid coating of the pH-MUC1-Pt NPs. (a) glacial acetic acid, maleic anhydride, (b) t-butyl carbazide, DCC, DCM, (c) 20%TFA in DCM, (d) CHCl_3_, Et_3_N, (e) 0.15M TFA in CHCl_3_; Figure S8: Monitoring of hydrazone bond formation via ^1^H-NMR. Disappearance aldehyde proton (top) vs. no signal in the final product (bottom); Figure S9: Determination of IC_50_ values for Apt-PLGA-Pt NPs [M] in CP70 cells; Figure S10: Determination of IC_50_ values for Apt-PLGA-Pt NPs [M] in SKOV-3 cells; Figure S11: Determination of IC_50_ values for Apt-PLGA-Pt NPs [M] in OVCAR-3 cells; Figure S12: Determination of IC_50_ values for Apt-PLGA-Pt NPs [M] in ES-2 cells; Figure S13: Determination of IC_50_ values for Apt-PLGA-Pt NPs [M] in OV-90 cells; Figure S14: Determination of IC_50_ values for Apt-PLGA-Pt NPs [M] in A2780 cells; Figure S15: Determination of IC_50_ values for Apt-PLGA-Pt NPs [M] in TOV-21G cells; Figure S16: Human to mouse dose conversion equation; Video S1: CP70_NP uptake.

## Abbreviations

NP	nanoparticle
Apt	aptamer
PLGA	poly(lactic)co-(glycolic) acid
PEG	polyethylene glycol
AAS	atomic absorption spectroscopy
TLC	thin layer chromatography
DLS	dynamic light scattering
TEM	transmission electron microscopy
FBS	fetal bovine serum
