# Supplementary material for: Pt(II)-PLGA Hybrid in a pH-Responsive Nanoparticle System Targeting Ovarian Cancer
Source: Pharmaceutics. 2023 Feb 10;15(2):607. doi: 10.3390/pharmaceutics15020607 (PMC9961376; doi:10.3390/pharmaceutics15020607)
Supplement: Supplementary file 1 [file pharmaceutics-15-00607-s001.zip › Supplementary Figures.pdf]

Pt(II)-PLGA hybrid in a pH-responsive nanoparticle system targeting ovarian cancer.

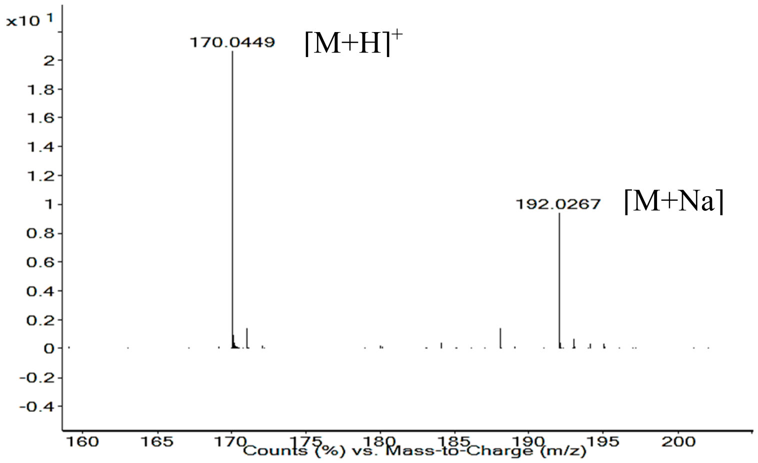

Figure S1. HRMS of (3-(2,5-dioxo-2,5-dihydro-1H-pyrrol-1-yl)propanoic acid) (**1**). HRMS-ESI: m/z $[M+H]^+$ calc. for $C_7H_7NO_4$: 170.0375; found: 170.0449.

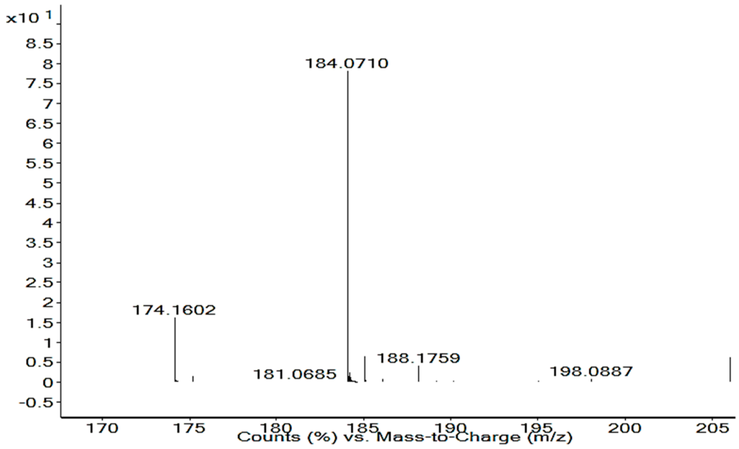

Figure S2. HRMS of (3-(2,5-dioxo-2,5-dihydro-1H-pyrrol-1-yl) propanehydrazide) (**3**). HRMS-ESI: m/z [M+H]+ calc. for $C_7H_9N_3O_3$: 184.0644; found: 184.0710.

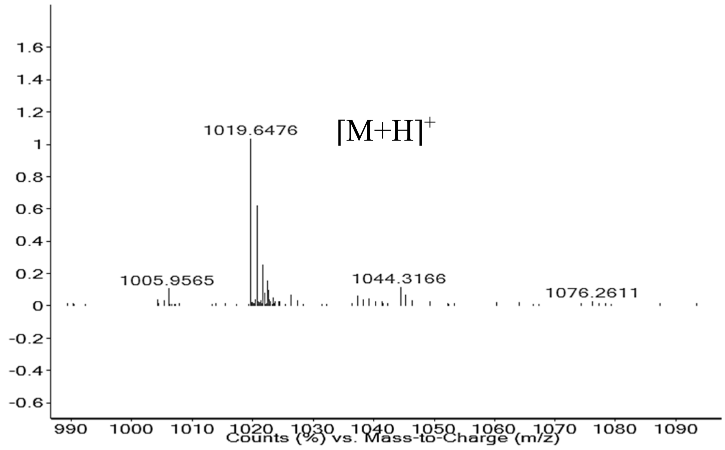

Figure S3. HRMS of (2,3-bis(stearoyloxy)propyl (2-((1-(3-hydrazineyl-3-oxopropyl)-2,5-dioxopyrrolidin-3-yl)thio)ethyl) phosphate) **(4)**. HRMS-ESI: m/z [M+H]+ calc. for $C_{51}H_{95}N_4O_{12}PS$: 1019.6405; found: 1019.6476.

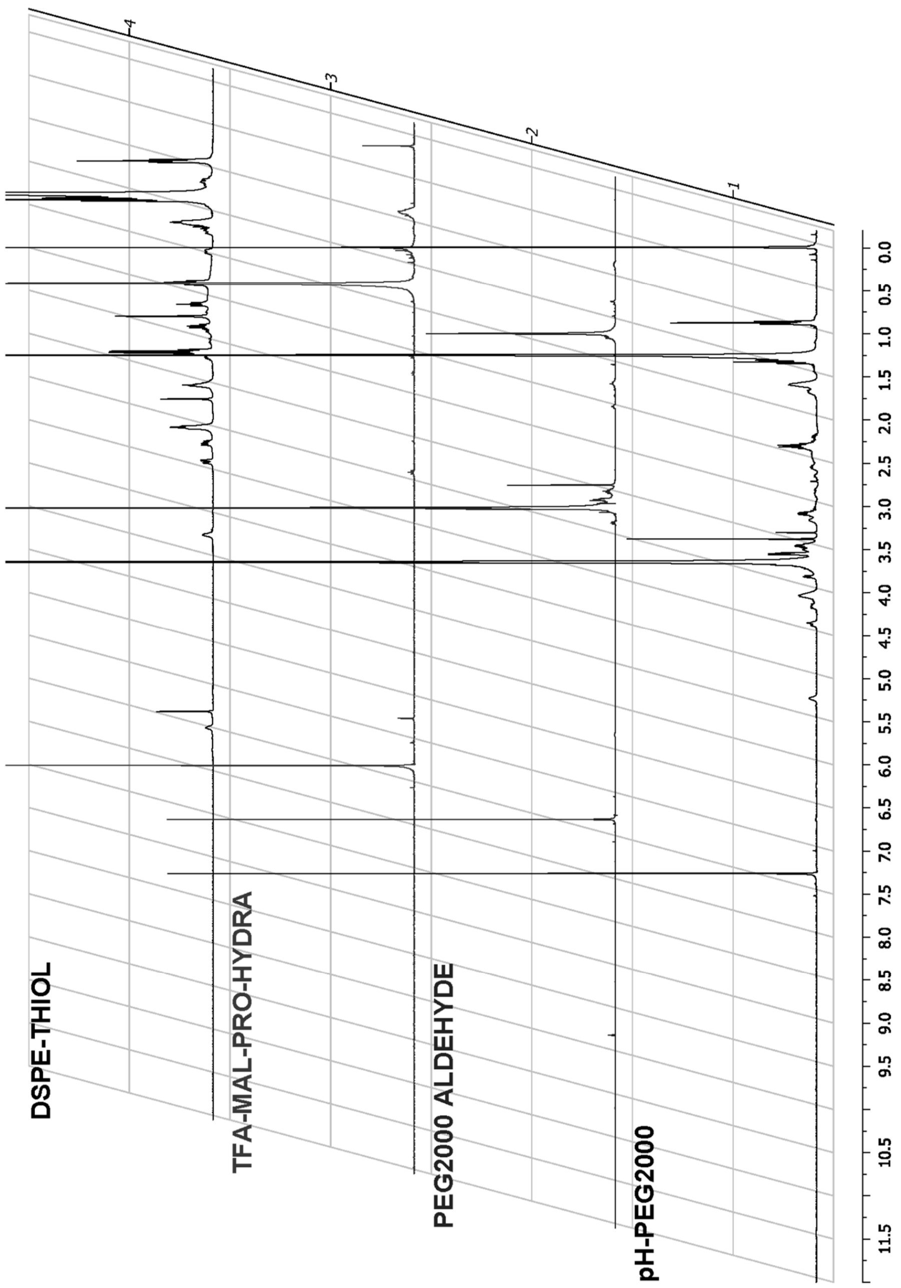

Figure S4. ^{1}H-NMR spectrum of: Top to bottom: DSPE-Thiol, 3-(2,5-dioxo-2,5-dihydro-1H-pyrrol-1-yl)propanehydrazide (3), PEG-2000 aldehyde and lipid-hydrazone-PEG2000 (5).

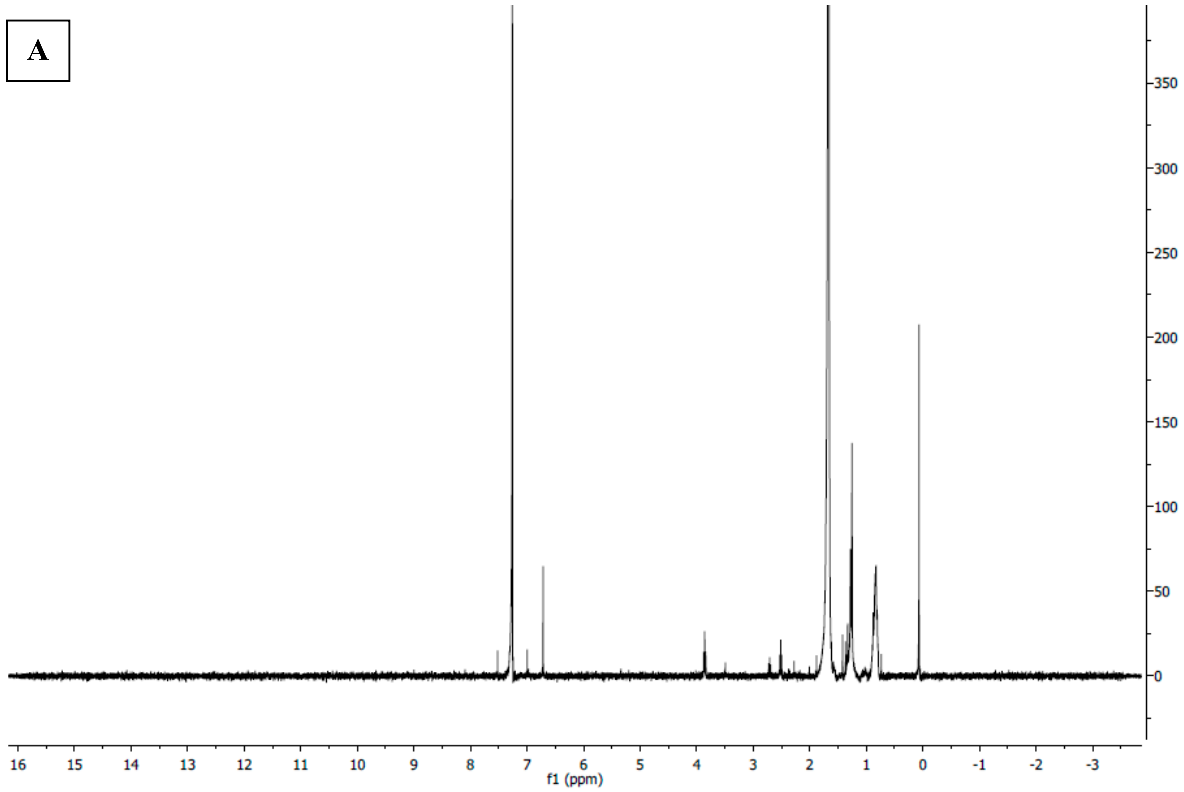
A
350
300
250
200
150
100
50
0
16 15 14 13 12 11 10 9 8 7 6 5 4 3 2 1 0 -1 -2 -3
f1 (ppm)

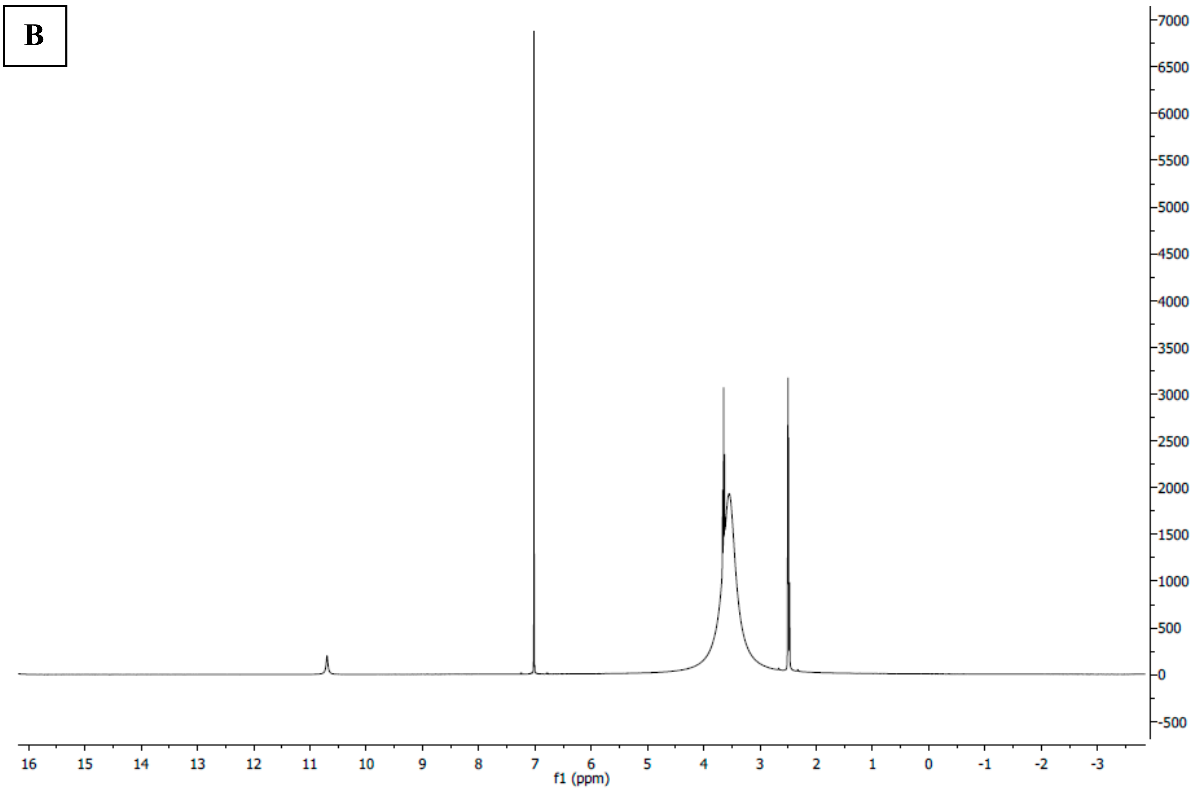
B
7000
6500
6000
5500
5000
4500
4000
3500
3000
2500
2000
1500
1000
500
0
-500
16 15 14 13 12 11 10 9 8 7 6 5 4 3 2 1 0 -1 -2 -3
f1 (ppm)

Figure S5. ^{1}H-NMR spectrum of: 3-(2,5-dioxo-2,5-dihydro-1H-pyrrol-1-yl)propanehydrazide (3) (A) in $CDCl_3$; (B) in DMSO-*d6*.

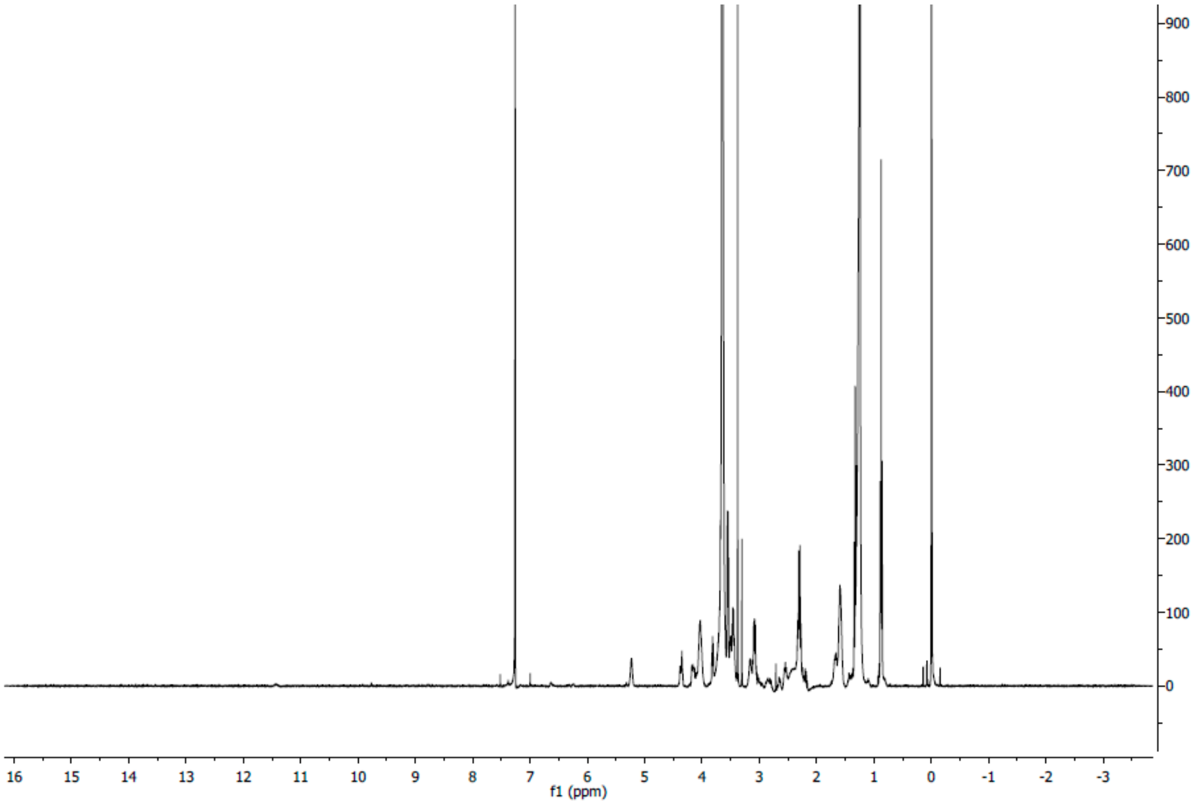

Figure S6. ^{1}H-NMR spectrum of: Lipid-Hydrazone-PEG2000 (5) in $CDCl_3$.

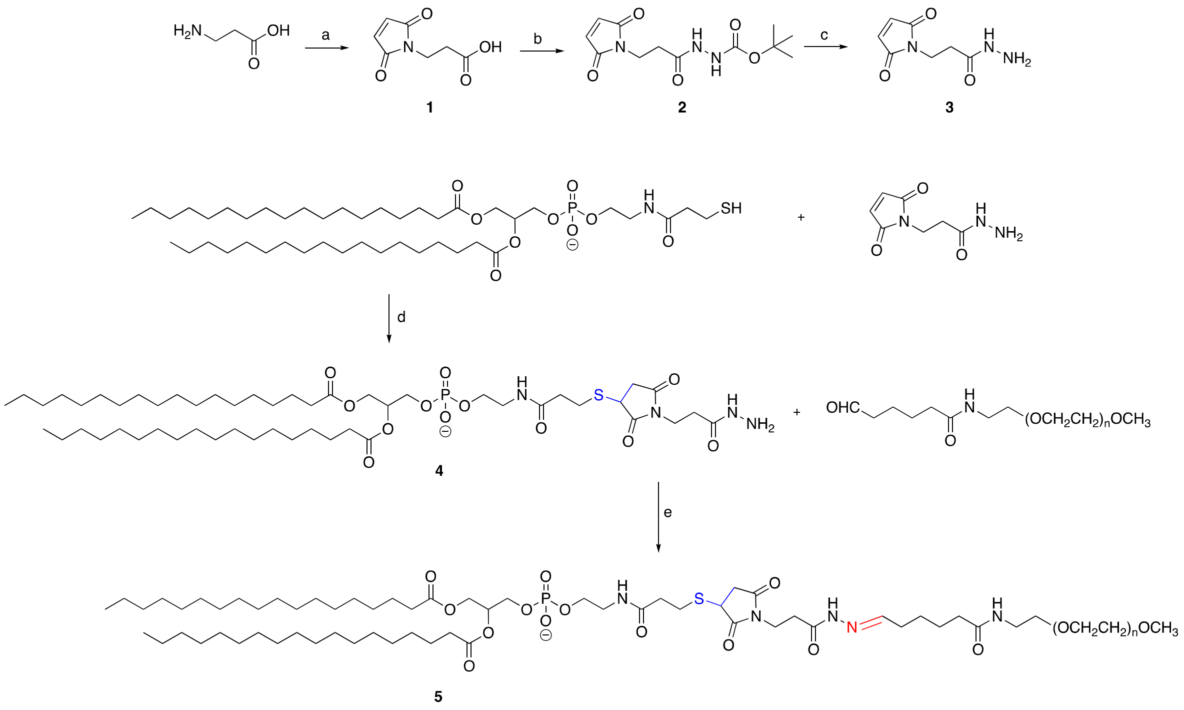

Figure S7. Schematic of the synthetic approach to pH-sensitive PEG2000-hydrazone-phospholipid coating of the pH-MUC1-Pt NPs. (a) glacial acetic acid, maleic anhydride, (b) *t*-butyl carbazide, DCC, DCM, (c) 20%TFA in DCM, (d) $CHCl_3$, Et_3N, (e) 0.15M TFA in $CHCl_3$.

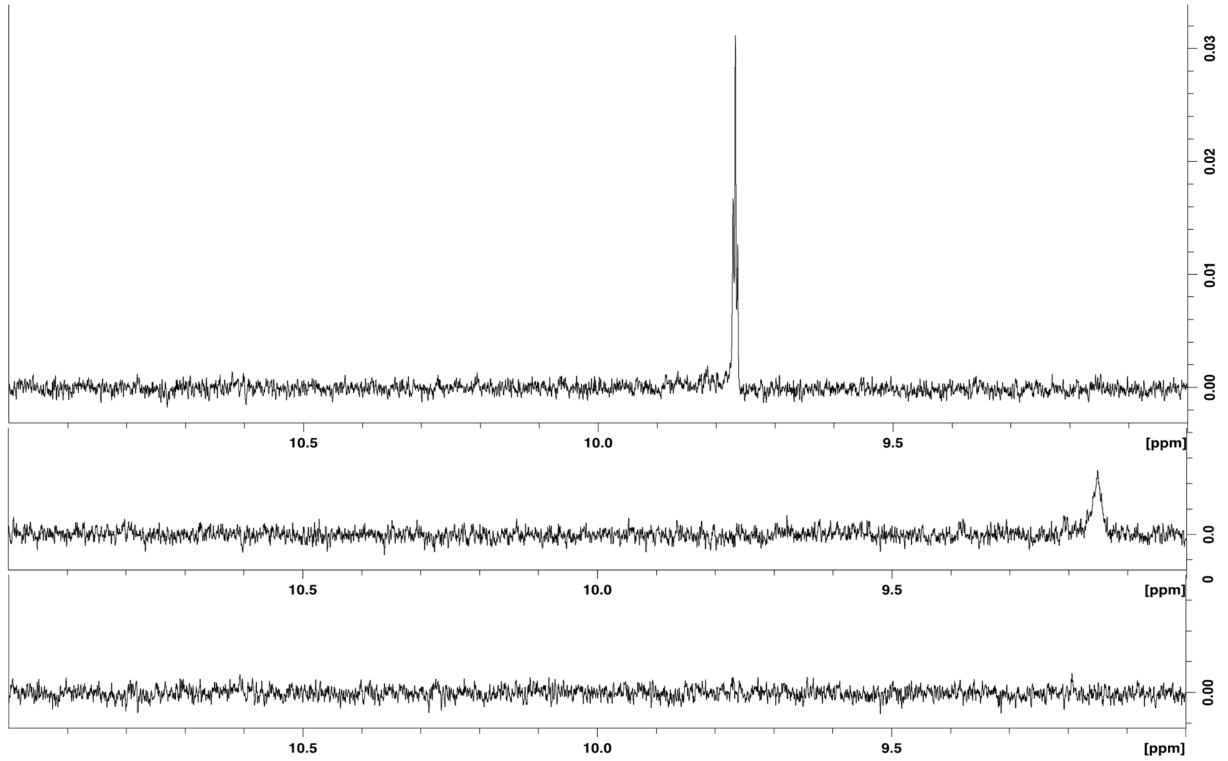

Figure S8. Monitoring of hydrazone bond formation via ^{1}H-NMR. Disappearance aldehyde proton (top) vs. no signal in the final product (bottom).

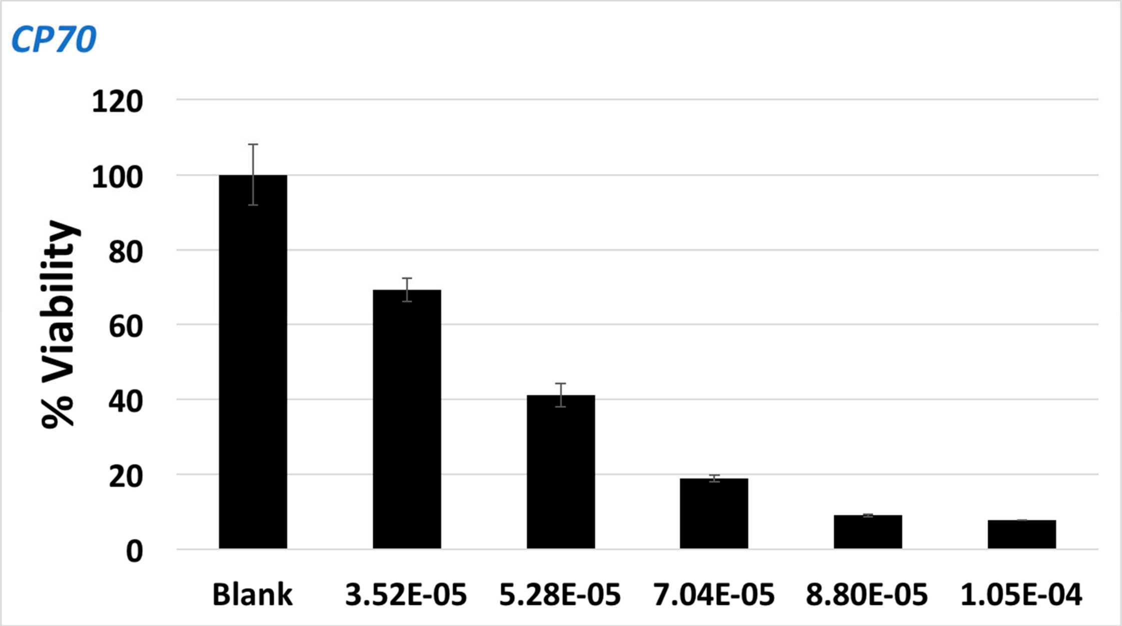

Figure S9. Determination of IC_{50} values for Apt-PLGA-Pt NPs [M] in CP70 cells.

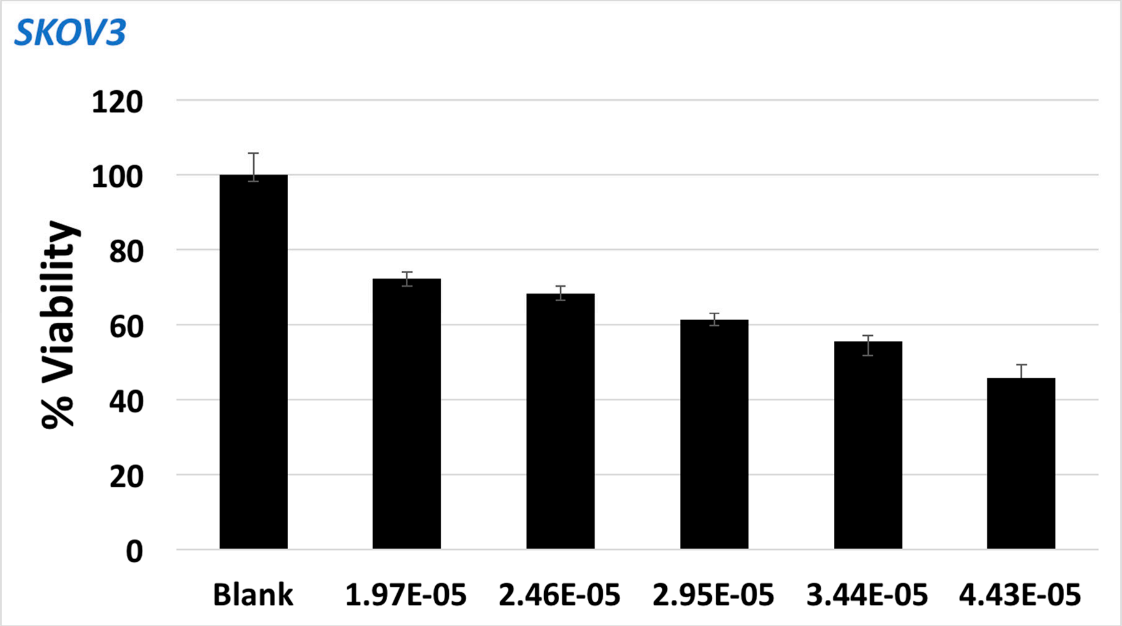

Figure S10. Determination of IC_{50} values for Apt-PLGA-Pt NPs [M] in SKOV-3 cells.

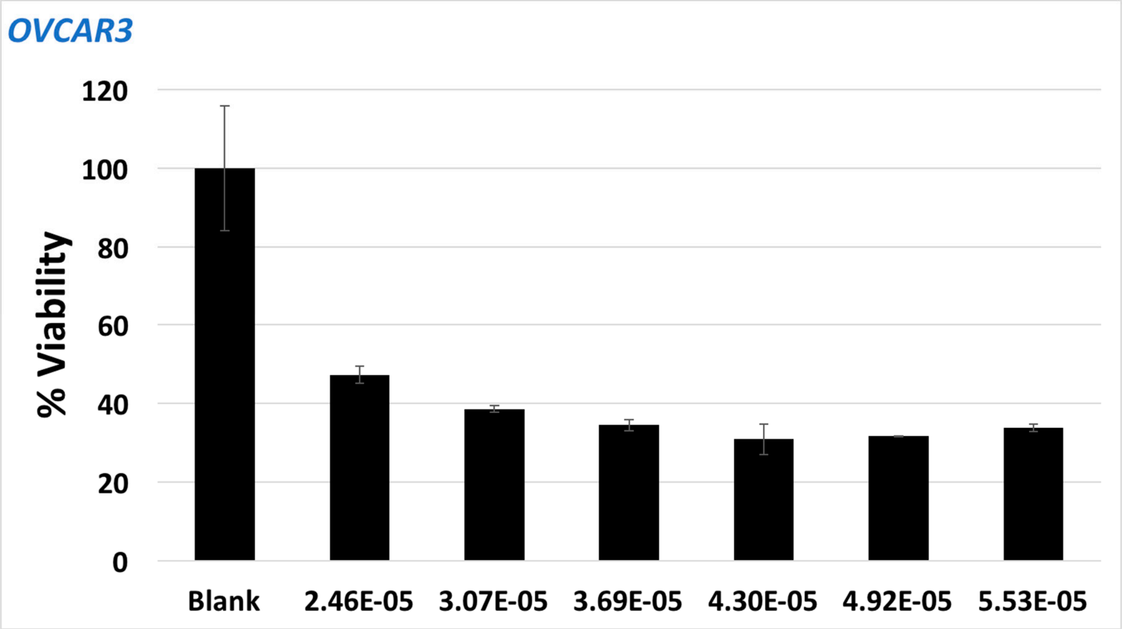

Figure S11. Determination of IC_{50} values for Apt-PLGA-Pt NPs [M] in OVCAR-3 cells.

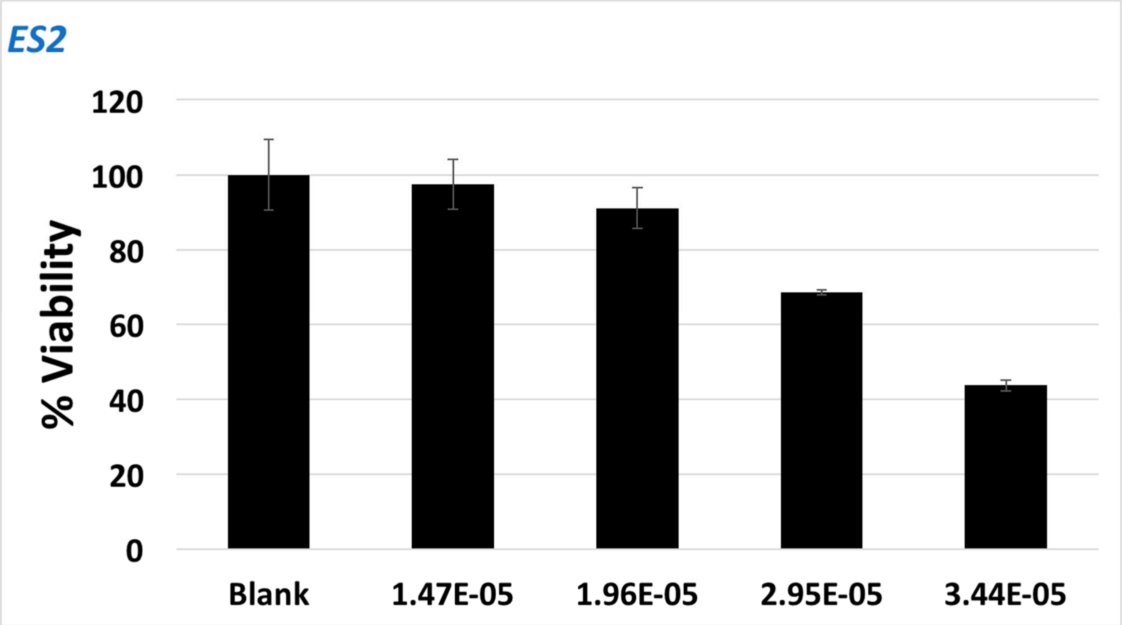

Figure S12. Determination of IC_{50} values for Apt-PLGA-Pt NPs [M] in ES-2 cells.

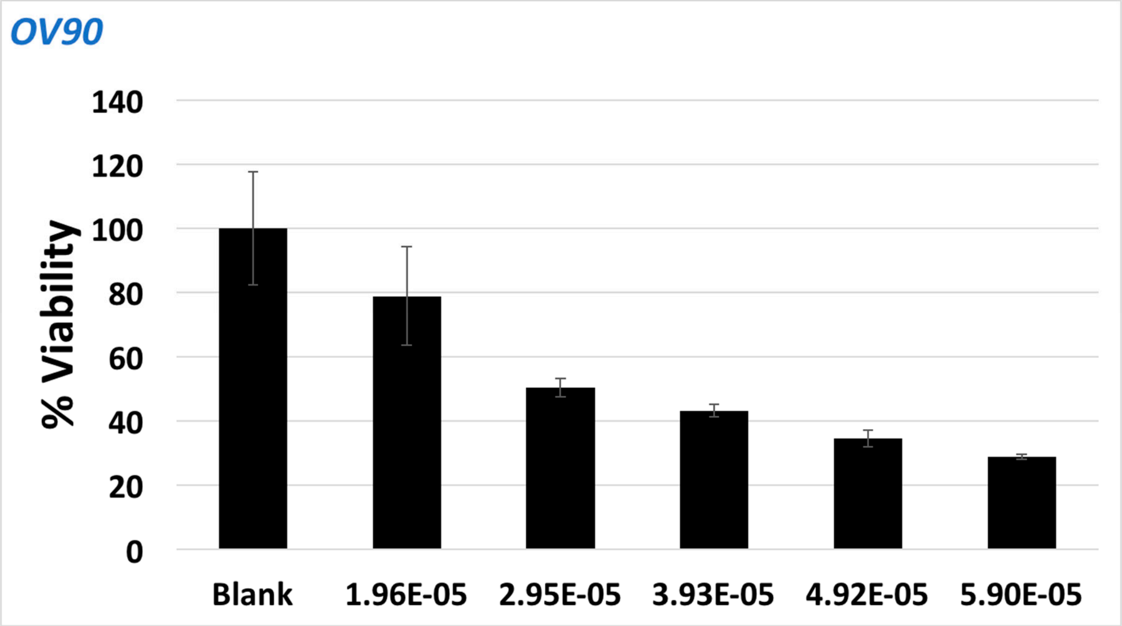

Figure S13. Determination of IC_{50} values for Apt-PLGA-Pt NPs [M] in OV-90 cells.

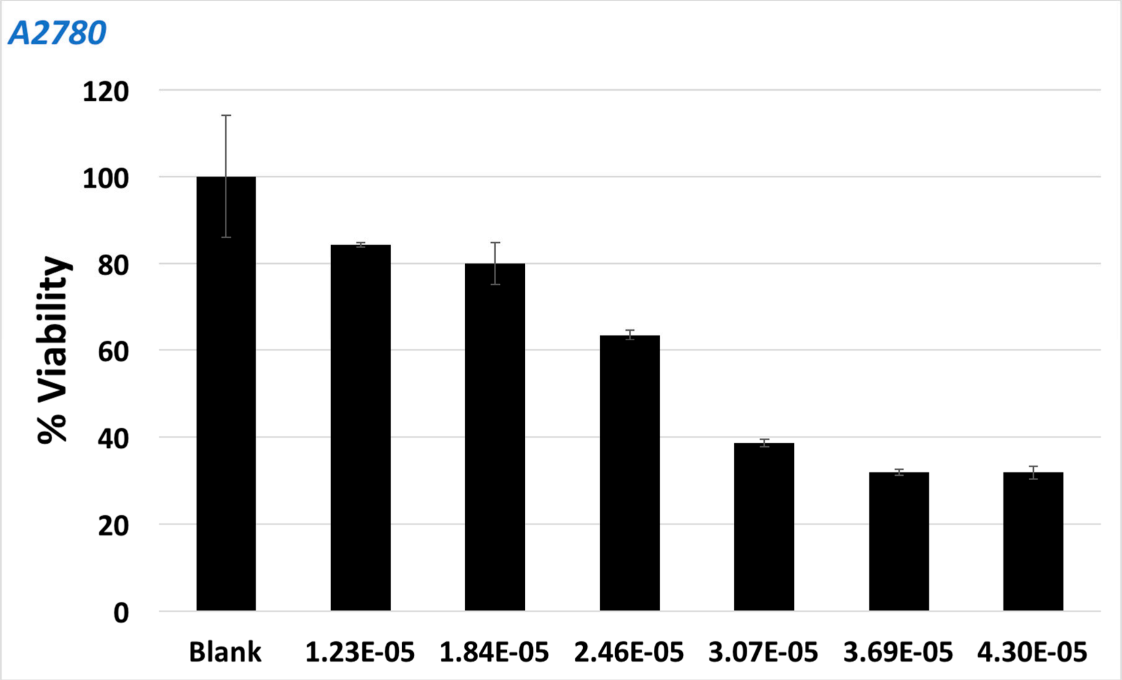

Figure S14. Determination of IC_{50} values for Apt-PLGA-Pt NPs [M] in A2780 cells.

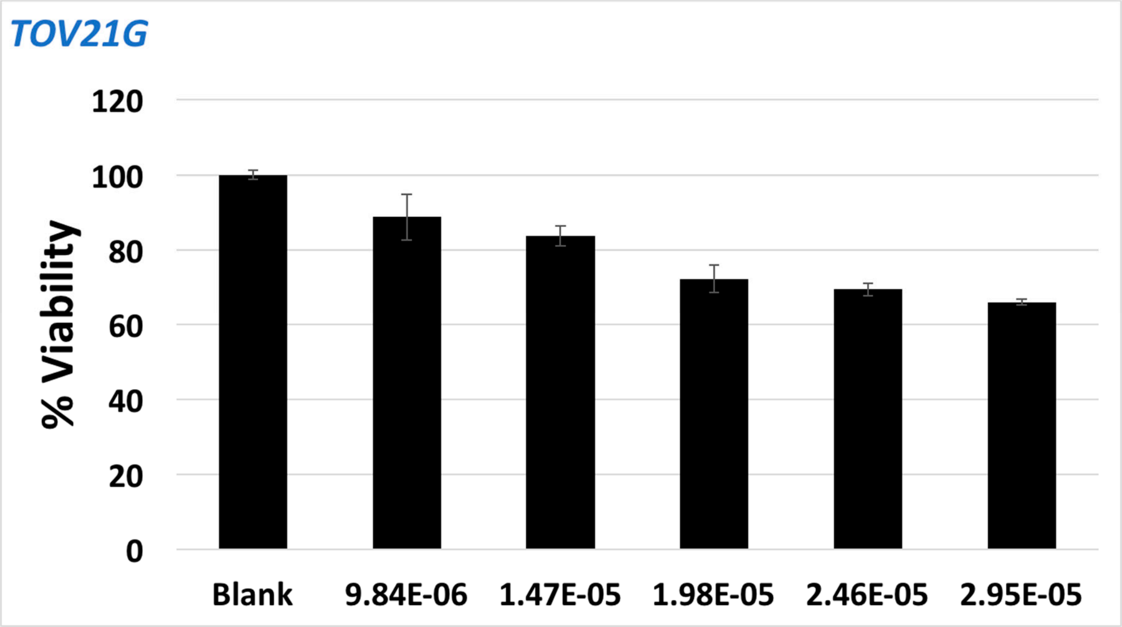

Figure S15. Determination of IC_{50} values for Apt-PLGA-Pt NPs [M] in TOV-21G cells.

HED (mg / kg) = Animal does (mg / kg) × (Animal K_m / Human K_m)

Mouse K_m= 3

Human K_m= 37

Figure S16. Human to mouse dose conversion equation.
